# The gut-lung axis in childhood asthma: from early-life programming to microbiome-informed precision medicine—a narrative review

**DOI:** 10.3389/fimmu.2026.1814901

**Published:** 2026-04-22

**Authors:** Miaojun Mo, Linlin Chen, Yi Wang, Xinyu Lin, Haiting Li, Binbin Chen, Junhui Yuan, Enfu Tao

**Affiliations:** 1 Department of Pediatrics, Wenling Maternal and Child Health Care Hospital, Wenling, Zhejiang, China; 2 Department of Neonatology and NICU, Wenling Maternal and Child Health Care Hospital, Wenling, Zhejiang, China; 3 Department of Delivery Room, Wenling Maternal and Child Health Care Hospital, Wenling, Zhejiang, China; 4 Department of Obstetrics, Wenling Maternal and Child Health Care Hospital, Wenling, Zhejiang, China

**Keywords:** childhood asthma, dysbiosis, early-life programming, gut-lung axis, microbial metabolites, microbiome, precision medicine, short-chain fatty acids

## Abstract

The gut-lung axis links early-life microbial programming to long-term respiratory health, offering a pivotal framework for understanding childhood asthma pathogenesis. This review synthesizes current evidence on how disruptions in microbial-immune crosstalk during critical developmental windows shape asthma susceptibility. Perinatal determinants—including maternal diet, delivery mode, antibiotic exposure, and breastfeeding—establish gut microbial communities that educate the developing immune system. Distinguishing itself from recent reviews, this review offers three novel contributions: (i) an integrated multi-omics framework linking early-life microbial maturation trajectories to specific asthma endotypes; (ii) a systematic synthesis of the molecular mechanisms by which microbial metabolites—including short-chain fatty acids, tryptophan derivatives, and bile acids—orchestrate gut-lung immune crosstalk; and (iii) a clinically actionable precision medicine algorithm that translates multi-omics profiling into personalized risk prediction, endotype-driven therapy selection, and targeted preventive strategies. Dysbiosis, characterized by delayed microbial maturation and depletion of short-chain fatty acid-producing taxa, compromises epithelial barrier integrity and skews immune homeostasis toward pro-allergic type-2 responses. Microbial metabolites, particularly short-chain fatty acids (acetate, propionate, butyrate) and tryptophan derivatives (indole-3-lactic acid, indole-3-propionic acid), serve as key molecular mediators that regulate regulatory T cells differentiation, reinforce mucosal barriers, and modulate distal airway inflammation. Microbial signatures correlate with specific asthma endotypes, offering opportunities for patient stratification. We critically evaluate emerging microbiome-targeted interventions—including strain-specific probiotics, prebiotics, postbiotics, and fecal microbiota transplantation—highlighting both therapeutic promise and the need for rigorous, well-powered clinical trials. Integrating multi-omics microbial profiling with host genetics and clinical phenotyping holds potential for microbiome-informed precision medicine, enabling personalized risk prediction, endotype-driven therapy selection, and novel preventive strategies targeting the gut-lung axis from the earliest stages of life.

## 1 Introduction

Asthma stands as the most common chronic disease among children globally, imposing a substantial and persistent burden on healthcare systems and the quality of life of millions of young individuals and their families (1, 2). Recent comprehensive analyses estimate that the global prevalence of current wheezing and ever asthma in individuals aged 5–69 years was approximately 11.5% and 9.8%, respectively, translating to hundreds of millions of affected persons worldwide (3). This condition is characterized by significant heterogeneity in its clinical presentation, underlying immunological mechanisms, and response to therapy (2, 4, 5). Epidemiological trends reveal a complex picture; while some data suggest a potential decrease in severe asthma symptoms among adolescents in some settings, the overall prevalence remains high with increases observed in certain regions and demographic groups (6–8). The Global Burden of Disease studies consistently rank asthma as a leading cause of years lived with disability, underscoring its pervasive impact on population health (9).

The clinical syndrome of asthma is now understood not as a single disease but as a spectrum of disorders, or endotypes, driven by distinct pathophysiological pathways (2, 4). A traditional immunological paradigm centers on type 2 (T2)-high inflammation, mediated by cytokines such as interleukin-4 (IL-4), IL-5, and IL-13, which leads to eosinophilia, IgE production, and bronchial hyperresponsiveness (4). However, a significant proportion of patients, particularly those with severe or corticosteroid-resistant disease, present with non-T2 or T2-low signatures, often associated with neutrophilic inflammation and different triggers (4, 10). This heterogeneity complicates management and highlights the limitations of a one-size-fits-all therapeutic approach. Despite significant advances in pharmacotherapy, including the refinement of inhaled corticosteroid strategies and the introduction of targeted biologic agents as reflected in updated guidelines like GINA 2021, challenges in disease control persist (11, 12).

This clinical and therapeutic complexity has catalyzed a profound shift in asthma research, moving beyond the airways to investigate systemic and developmental origins of the disease. A dominant theme in contemporary investigation is the role of early-life environmental exposures in programming long-term immune function and disease susceptibility (1, 10, 13). Concurrently, the last decade has witnessed a revolution in our understanding of the human microbiome and its integral role in educating and regulating the host immune system (14, 15). It is at the intersection of these two research frontiers—early-life programming and host-microbiome symbiosis—that the concept of the gut-lung axis has emerged as a critical framework for understanding asthma pathogenesis (16–18).

The gut-lung axis represents a bidirectional communication network wherein the intestinal microbiota and its metabolic products systemically influence immune homeostasis and inflammatory responses in the distal lungs, and vice versa (18–20). In health, the microbiome is fundamental to systemic immune and metabolic homeostasis, with early-life colonization playing a particularly prominent role in shaping the developing immune system (1, 14). Disruptions to this delicate developmental process—termed dysbiosis—can predispose infants to atopic diseases, including asthma (1, 14, 17). This review aims to synthesize the rapidly evolving evidence on the gut-lung axis in childhood asthma. We will explore the fundamentals of microbial-immune homeostasis, delineate the critical window of early-life microbiome development and its perinatal determinants, characterize the dysbiotic signatures associated with asthma, and elucidate the molecular mediators, such as microbial metabolites, that facilitate cross-talk. Furthermore, we will examine how environmental and host factors shape this axis and discuss the promising translational potential of microbiome-targeted interventions. Finally, we will consider the path toward microbiome-informed precision medicine, addressing the heterogeneity of asthma to improve prevention, stratification, and treatment outcomes for children worldwide (16, 17). By integrating recent advances in multi-omics, microbial metabolites, and precision medicine, this review aims to provide a comprehensive and clinically relevant framework for understanding and targeting the gut-lung axis in childhood asthma.

## 2 Fundamentals of the gut-lung axis and microbial-immune homeostasis

The gut-lung axis conceptualizes a sophisticated, bidirectional communication network where the intestinal microbiota and its metabolic products exert systemic influence on immune homeostasis and inflammatory responses in the lungs, and where pulmonary immune status and inflammation can, in turn, affect the gut environment (21). This cross-talk is fundamentally orchestrated by the host’s microbiota, a vast community of bacteria, fungi, viruses, and archaea residing on mucosal surfaces, with the gastrointestinal tract harboring the most dense and diverse consortium (14, 22). In health, a state of symbiotic equilibrium is maintained where the microbiota contributes to vital host functions, including nutrient metabolism and, most critically, the proper education and regulation of the immune system (23–25). The immune system, in return, deploys mechanisms to contain the microbiota within the intestinal lumen and preserve barrier integrity, thereby preventing inappropriate inflammation (24, 26). This dynamic, mutualistic interaction begins prenatally and is cemented in early postnatal life, setting the immunological tone that can confer long-term protection against or predisposition to inflammatory diseases such as asthma (14, 27–29).

The early-life period represents a critical window for microbial-immune imprinting, where initial colonizers and environmental exposures instruct the developing immune system (28, 29). Maternal microbial and dietary factors during pregnancy, followed by delivery mode, infant diet (particularly breastfeeding), and antibiotic use, sequentially shape the neonatal gut ecosystem (14, 23). Breastfeeding, for instance, provides human milk oligosaccharides (HMOs) that selectively nourish beneficial bacteria like *Bifidobacterium infantis*, which are equipped with specialized gene sets for their utilization (30). The metabolic activity of such pioneer bacteria is not merely nutritive but immunologically instructive. Supplementation with *B. infantis* EVC001 in breastfed infants was shown to suppress intestinal pro-inflammatory T helper 2 (Th2) and Th17 cytokines while inducing interferon-β, with the microbial metabolite indole-3-lactic acid (ILA) identified as a key mediator that upregulated the immunoregulatory protein galectin-1 in immune cells (30). This exemplifies how specific commensal microbes and their metabolites can directly program immune responses during a vulnerable developmental phase. The maturation of a balanced immune repertoire relies on continuous exposure to microbial signals, which promote the expansion and functional specialization of immunosuppressive cell populations, notably regulatory T cells (Tregs) (31, 32).

Intestinal Tregs are paramount in maintaining mucosal tolerance and systemic immune homeostasis. While a subset originates from the thymus, a significant proportion is induced peripherally in gut-draining lymph nodes from conventional CD4+ T cells upon exposure to dietary and microbial antigens (31). These cells are essential for restraining aberrant immune activation. Experimental evidence underscores the lasting importance of Tregs generated during the peri-weaning period; their specific ablation after weaning, but not before, led to elevated systemic IgE and IL-13 levels and impaired tolerance induction in adulthood (32). However, it is important to acknowledge that these findings derive from a murine model, and direct extrapolation to human developmental immunology requires caution due to species-specific differences in immune maturation timelines. The peri-weaning period in mice does not directly correspond to specific human developmental stages, underscoring the need for human validation studies to confirm the clinical relevance of these observations. This highlights that the immune system’s education by microbiota is not a static event but a process requiring sustained microbial dialogue to maintain a regulatory phenotype.

The molecular mediators of the gut-lung dialogue are largely microbial metabolites derived from the fermentation of dietary components. Short-chain fatty acids (SCFAs)—primarily acetate, propionate, and butyrate—produced by commensal bacteria from dietary fiber, are among the most well-studied immunomodulatory metabolites (22, 25, 33). SCFAs signal through specific G protein-coupled receptors (GPRs) such as GPR43 (FFAR2) and GPR41 (FFAR3), and also inhibit histone deacetylases (HDACs), leading to epigenetic reprogramming of immune cells (33, 34). Through these mechanisms, SCFAs enhance gut epithelial barrier integrity, promote the differentiation and function of colonic and systemic Tregs, and dampen the proliferation and effector functions of pro-inflammatory Th2 cells (22, 33, 35). Beyond SCFAs, metabolites from tryptophan catabolism, such as indole derivatives, engage the aryl hydrocarbon receptor (AhR) on epithelial and immune cells, reinforcing barrier function and promoting anti-inflammatory responses (22, 35).

The integrity of the epithelial barrier is the foundational physical and immunological determinant of host-microbe interaction at all mucosal sites. A healthy, intact barrier segregates the luminal microbiota from host immune cells, preventing translocation and uncontrolled immune activation (26, 36). Maintaining barrier homeostasis is a crucial function of the microbiota and a key endpoint of beneficial microbial metabolites like SCFAs and AhR ligands (26, 35). Disruption of this homeostatic balance—through dysbiosis, barrier dysfunction, or pathogen colonization—can lead to immune dysregulation and contribute to asthma pathogenesis.

## 3 The critical window: early-life microbiome development and perinatal determinants of asthma susceptibility

**Figure 1:**
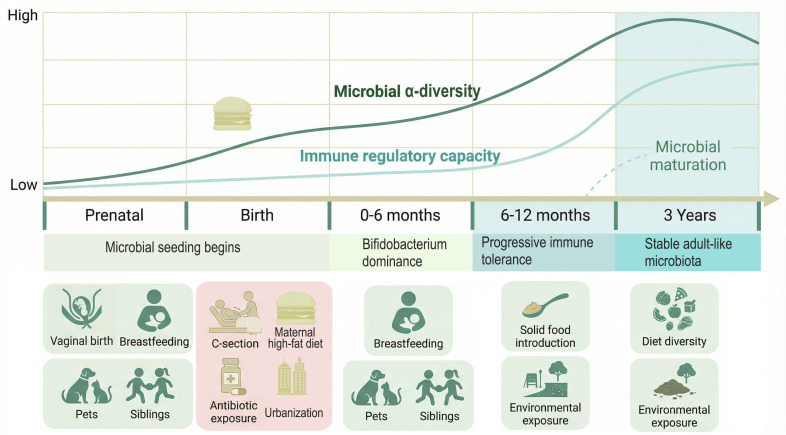
The critical window of early-life gut-lung axis development. Schematic timeline illustrating the parallel development of the gut microbiota and immune system from the prenatal period through early childhood. The upper panel shows the trajectory of microbial α-diversity (dark green line) and immune regulatory capacity (light green line) over time. Key microbial colonization and immune maturation events are indicated, including *Bifidobacterium* dominance during exclusive breastfeeding (0–6 months), progressive microbial diversification following solid food introduction (6–12 months), and establishment of a stable adult-like microbiota by 3 years of age. Immune tolerance develops progressively throughout this period, with critical reinforcement during the peri-weaning phase. The lower panel depicts major perinatal and postnatal determinants that shape this co-development, categorized as protective factors (green background, e.g., vaginal delivery, breastfeeding, siblings, pet exposure) or risk factors (pink background, e.g., cesarean section, prenatal and infant antibiotic exposure, maternal high-fat diet, urbanization). This critical window represents a dynamic interface where genetic predisposition and environmental exposures collectively program long-term respiratory health outcomes. Abbreviations: α-diversity, alpha diversity; C-section, cesarean section.

The developmental trajectory of the immune system and its functional setpoints are profoundly influenced by early-life microbial exposure, establishing a period of heightened plasticity that is now recognized as a critical window for asthma susceptibility (37, 38). During this time, which spans from the prenatal period through the first few years of life, the parallel and interactive maturation of the microbiome and immune system occurs (39, 40). Disruptions to this co-development, driven by a constellation of perinatal and postnatal factors, can divert immune programming towards a state of dysregulation, thereby increasing the risk of childhood asthma (41). This chapter synthesizes evidence on how early microbial seeding, maturation, and environmental exposures during this sensitive period interact to shape long-term respiratory health outcomes. This developmental trajectory, spanning from the prenatal period through early childhood, is schematically illustrated in **Figure 1**, which depicts the parallel maturation of microbial diversity and immune regulatory capacity alongside key perinatal determinants that shape long-term respiratory health outcomes.

The foundations of the gut-lung axis are laid even before birth. The maternal microbiome and her immune status during pregnancy exert a priming effect on the offspring’s future immune responses and microbial colonization patterns (42, 43). Maternal dietary patterns, for instance, are associated with distinct maternal and neonatal gut microbiota compositions, with implications for infant immune development (44, 45). High intake of saturated and monounsaturated fats has been linked to specific microbial profiles, while fibre intake may support greater microbial diversity (44, 45). Furthermore, the maternal immune milieu, such as the balance between IFN-γ and IL-13 during the third trimester, can shape the neonatal epigenome and trained innate immunity, which in turn is linked to pathologic airway microbial colonization and later asthma (42). Perturbations such as antibiotic use during pregnancy are associated with offspring gut dysbiosis, intestinal barrier dysfunction, and a skewing of immune cells towards Th17 and RORγt^+^ Treg phenotypes, creating a pro-asthmatic state along the gut-lung axis (46, 47). This prenatal programming is reflected in the newborn’s blood metabolome at birth, where a cesarean section-associated metabolic signature—characterized by perturbations in tryptophan, bile acid and phenylalanine metabolites—is linked to altered cord blood Treg frequency and an increased risk of childhood asthma (48).

The initial microbial seeding during and after birth is a pivotal event. While the exact magnitude of *in utero* microbial transfer remains debated, robust evidence confirms that the mother is a primary source for the infant’s founding microbiota across multiple body sites (38, 49). Mode of delivery is a major determinant of this vertical transmission, with vaginally delivered infants acquiring microbes resembling the maternal vaginal and fecal microbiota, whereas cesarean-delivered infants exhibit a microbiota more akin to maternal skin and the hospital environment (49, 50). Importantly, the asthma risk associated with cesarean section appears to be mediated by the persistence of a cesarean-like microbial signature at one year of age, rather than the delivery mode itself, highlighting that appropriate gut microbiota maturation can mitigate the risk (51). Recent evidence suggests that while cesarean section reduces direct seeding from the maternal fecal microbiota, compensatory routes such as increased colonization from breast milk microbes may occur, ensuring functional transfer (49). Notably, the common practice of administering antibiotic prophylaxis before cesarean section appears to have only a limited additional impact on the infant gut microbiome compared to the dominant influence of feeding mode (52).

Beyond initial seeding, the pace and pattern of microbial community maturation in infancy are critical. A landmark finding is that delayed gut microbiota maturation during the first year of life appears to be a hallmark of pediatric allergic diseases, including asthma (39, 53). This maturation can be quantified as “estimated microbiome age,” with more advanced maturation (a higher estimated microbiome age) associated with protection, as demonstrated in the context of the farm environment’s asthma-protective effect (53). Conversely, both accelerated maturation very early in life (at 3–6 months) and delayed maturation later (at 1 year) have been associated with increased asthma risk, suggesting there are specific, age-dependent normative trajectories (54). This impaired maturation is characterized by functional imbalances, including compromised mucous integrity, elevated oxidative activity, and reduced secondary fermentation, which mediate the link between microbiota structure and allergic disease (39). Thus, the ecological succession towards a stable, diverse, and functional adult-like microbiota, rather than merely the presence of specific taxa at a single time point, is a key determinant of health (50, 55).

A wide array of modifiable environmental exposures during this critical window can influence microbial maturation and asthma risk. Antibiotic exposure in infancy remains one of the most significant and well-characterized risk factors (41, 56). Population-level data show a strong temporal correlation between decreasing antibiotic prescribing in infancy and declining childhood asthma incidence (56). Mechanistic studies in humans indicate the gut microbiota mediates this relationship, with antibiotic exposure reducing α-diversity and altering community structure (56). Animal models corroborate this, showing that early-life antibiotic exposure, and even the transfer of an antibiotic-perturbed microbiota to germ-free mice, can heighten allergic airway responses in the next generation (46, 57). The risk appears dose-dependent, with higher cumulative exposure, use of macrolides, and exposure very early in life conferring greater risk (47, 58). Conversely, traditional asthma-protective factors such as having siblings, particularly with a smaller age gap to the nearest older sibling, are strongly associated with distinct and protective microbiota profiles in the gut and airways during infancy (59, 60). Prenatal exposure to pets, especially dogs, is also linked to increased infant gut microbial diversity and enrichment of specific taxa like *Fusobacterium* and *Lachnospiraceae*, offering a potential microbial mechanism for the observed protective effect against allergy (61, 62). Breastfeeding influences microbial composition, enriching for *Bifidobacterium* and other beneficial taxa, and its protective effect against asthma is significantly mediated by infant gut microbiota maturation (54, 63, 64). Maternal diet during lactation, though less studied, is a plausible modulator of the milk microbiota and, consequently, the infant gut microbiome (65).

Finally, this early-life microbial programming does not occur in a genetic vacuum. Host genetics interact with environmental exposures to shape the microbiome and asthma risk. For example, the asthma-associated risk conveyed by the infant gut virome is modulated by a host TLR9 gene variant (66). The 17q12–21 asthma risk locus and gut microbial maturation exert independent and additive effects on asthma risk, indicating distinct pathways that can converge (54). Even the human milk microbiota, which is associated with childhood asthma and atopy, shows links to maternal genetic loci, illustrating the complex interplay between host heredity and the microbial environment transmitted to the infant (67). These interactions underscore that the critical window represents a dynamic interface where genetic predisposition, microbial succession, and environmental exposures collectively determine the trajectory towards respiratory health or disease.

## 4 Dysbiosis and immune dysregulation: the microbial signature of childhood asthma

Building on the homeostatic mechanisms described in the previous section, this section examines how disruption of microbial-immune equilibrium— dysbiosis—contributes to asthma pathogenesis. While the previous section focused on the establishment and maintenance of immune tolerance through commensal microbes and their metabolites, here we explore how shifts in microbial composition and function lead to immune dysregulation and promote allergic airway inflammation. Asthma is no longer viewed as a single disease but as a spectrum of endotypes, broadly categorized into T2-high and non-T2 (or T2-low) phenotypes (68). The gut-lung axis influences both through distinct mechanisms, reflecting the complex interplay between local airway microbial alterations and systemic immunomodulatory effects of the gut microbiota. These airway microbial alterations are directly linked to asthma pathogenesis (14, 21). T2-high asthma is characterized by eosinophilic inflammation driven by cytokines like IL-4, IL-5, and IL-13, where a lack of early microbial exposure or SCFAs may fail to adequately restrain T2 differentiation (33, 69). Non-T2 asthma involves other immune pathways, including neutrophilic inflammation and roles for innate immune cells like macrophages and mast cells, whose maturation and function are also modulated by microbial signals (21, 68). Parallel to these local effects, the gut microbiota exerts a profound systemic immunomodulatory influence, shaping the bone marrow output of immune cell progenitors and the tone of circulating immune cells, thereby determining the inflammatory potential of the entire respiratory system (14, 23). Understanding these fundamental mechanisms of microbial-immune homeostasis and cross-talk is essential for deciphering how disruptions along the gut-lung axis contribute to the inception and persistence of childhood asthma.

Disruption of the sustained microbial dialogue required for immune homeostasis can skew immune differentiation. In the context of allergy and asthma, this often manifests as an imbalance between pro-inflammatory Th2 cells and counter-regulatory Tregs or Th1 cells (69, 70). The gut microbiota influences this balance by modulating the activity of dendritic cells and other antigen-presenting cells, thereby shaping the T-cell response landscape in distal sites like the lungs (69). Consistent with the physiological roles of SCFAs outlined in Section 2, dysbiosis-induced depletion of SCFAs-producing taxa compromises these protective mechanisms. Insufficient dietary fiber intake, common in Western diets, further exacerbates SCFAs deficiency and is proposed as a significant contributor to the rising incidence of mucosal inflammatory diseases, including asthma, offering an alternative or complementary perspective to the hygiene hypothesis (33, 35). The consequent reduction in SCFAs-mediated Treg differentiation and barrier reinforcement shifts the immune set point toward pro-allergic type-2 responses, as reflected in the elevated Th2 cytokines and impaired regulatory function characteristic of asthmatic children.

The healthy epithelial barrier serves to segregate luminal microbiota from host immune cells, preventing inappropriate inflammation (26, 36). The “Epithelial Barrier Theory” provides a unifying framework, positing that the modern exposome—encompassing environmental toxins, pollutants, detergents, processed foods, and microplastics—damages the epithelial lining of the skin, respiratory tract, and gut (36, 71, 72). This damage leads to “epithelitis”, a state of chronic periepithelial inflammation, translocation of microbes and their products, dysbiosis, and ultimately, the initiation or exacerbation of chronic inflammatory diseases, including allergic asthma (36, 72). A leaky gut barrier, for example, may allow increased systemic dissemination of microbial components or metabolites that can prime distal organs, like the lungs, for exaggerated inflammatory responses to allergens (71, 72). In the airways, early-life colonization patterns deviate from homeostatic microbial succession. Whereas healthy microbial communities typically favor commensals that promote immune tolerance, dysbiosis is characterized by expansion of potentially pathogenic taxa. For example, neonatal colonization of the airways with specific pathogenic bacteria such as *Streptococcus pneumoniae*, *Haemophilus influenzae*, and *Moraxella catarrhalis* is consistently associated with a markedly increased risk of persistent wheeze and asthma in early childhood (73, 74). This association, replicated across cohorts using both culture and sequencing methods, appears most potent for asthma onset before school age, with effects on exacerbations and inflammatory markers like blood eosinophils and TNF-α diminishing by adolescence (73, 74). Furthermore, the developmental trajectory of the nasopharyngeal microbiome in the first two years is informative; an early *Staphylococcus*-dominant profile is linked to later asthma, while the predominance of *Moraxella* during viral wheezing illnesses is associated with persistent disease (75). Early-life viral infections themselves are intricately linked to the airway ecosystem, triggering specific immune responses and shaping subsequent microbiota dynamics that can influence susceptibility to recurrent respiratory infections (76, 77). Notably, children who later develop asthma exhibit a reduced regulatory immune response, characterized by lower levels of IL-10, during early-life viral episodes (77).

Building on the concept of a critical developmental window, a distinct microbial signature emerges in children who develop asthma. This signature is characterized by dysbiosis and is intricately linked to dysregulated immune responses that underpin asthma pathogenesis. The evidence points not to a single pathogen but to specific, reproducible shifts in microbial ecology across the gut-lung axis that are associated with increased risk, severity, and specific phenotypes of childhood asthma (78, 79). In older children with established asthma, airway dysbiosis persists, with specific profiles correlating with clinical features. For instance, sputum enrichment with genera like *Haemophilus* and *Neisseria* is associated with fixed airflow obstruction and mixed granulocytic inflammation (80). The respiratory microbiome’s role is dynamic and seasonally influenced; specific bacterial networks (e.g., *Streptococcus*- or *Staphylococcus*-dominant) interact with host transcriptional modules to significantly increase the odds of seasonal asthma exacerbations (81). Importantly, airway microbial dysbiosis in asthma is not confined to bacteria. The upper airway mycobiome exhibits significant alterations, where a higher relative abundance of the commensal fungus *Malassezia globosa* is associated with better asthma control and a lower risk of progression to severe exacerbation (82). Conversely, an expansion of the eukaryotic virome, including heightened abundances of cytomegalovirus and Epstein-Barr virus alongside a reduction of bacteriophages, characterizes the airways of asthmatic children and correlates with disease severity and exacerbation (83, 84). This viral and fungal dysbiosis indicates a broad ecological disruption within the respiratory ecosystem in asthma. These airway microbial alterations are directly linked to asthma pathogenesis (14, 21). However, the gut microbiota exerts a profound systemic immunomodulatory effect, influencing the bone marrow output of immune cell progenitors and the tone of circulating immune cells, thereby shaping the inflammatory potential of the entire respiratory system (14, 23). The heterogeneity of asthma—encompassing T2-high and non-T2 endotypes—reflects the complex interplay between these local and systemic microbial influences on immune development and function. Parallel to airway alterations, the gut microbiome of children with asthma exhibits profound and functional dysbiosis. A landmark finding is that delayed maturation of the gut microbiota in the first year of life is a universal hallmark of various pediatric allergic diseases, including asthma (39). This impaired maturation is characterized by a core set of functional imbalances, such as compromised mucosal integrity and elevated oxidative activity, which mediate the link between early-life microbiota and later asthma diagnosis (39). Taxonomically, childhood asthma is frequently associated with an increased relative abundance of *Proteobacteria* and a depletion of anaerobic *Firmicutes*, particularly members of the class *Clostridia* (79, 85). These shifts are not uniform and appear to relate to asthma phenotype; for example, non-allergic asthma may be associated with more pronounced gut bacterial alterations and a distinct metabolomic profile, including elevated histamine, compared to allergic asthma (85). The functional impact of gut dysbiosis is evident in its association with asthma morbidity. In children with established asthma, the gut microbiome and metabolome correlate with symptom frequency; higher wheeze proportion is associated with enrichment of genera like *Veillonella* and specific histidine pathway metabolites (86). Furthermore, the gut microbiota from asthmatic individuals can directly influence lung inflammation, as demonstrated in gnotobiotic mice, with enterotoxigenic *Bacteroides fragilis* (ETBF) identified as more prevalent in asthma and capable of exacerbating oxidative stress in the lungs during allergic inflammation (87). While gnotobiotic mouse models are valuable for establishing causal relationships, their inherent limitations must be acknowledged when translating findings to human asthma. Germ-free mice exhibit altered immune development that may not fully recapitulate human disease contexts. Therefore, findings from such models should be interpreted as mechanistic proof-of-concept requiring validation in human cohorts. Gut dysbiosis also intersects with other risk factors, such as race and co-morbid atopy, where differences in gut microbial composition, including lower abundance of certain *Bifidobacterium* species, are observed in Black children with food allergy who have a higher rate of asthma (88).

The microbial signatures of asthma are not merely associative but are mechanistically linked to the immune dysregulation that defines the disease. Dysbiosis disrupts the delicate balance between immune tolerance and inflammation. In the airways, colonization with pathogens like *Moraxella* and *Haemophilus* is linked to a neutrophilic inflammatory response and recurrent wheezing (89). This bacterial dysbiosis can be primed by viral infections; respiratory syncytial virus (RSV) infection in infancy is strongly associated with later asthma development and can disrupt established immune tolerance by skewing the T-helper cell balance towards a Th17 response and away from Tregs activity (90, 91). Conversely, episodic aspiration of certain oral commensals (e.g., *Prevotella*, *Veillonella*) can induce a prolonged, MyD88-dependent Th17 response in the lungs that may be protective against subsequent pathogen challenge, highlighting the context-dependent nature of host-microbe interactions (92). The gut microbiome exerts systemic immunomodulatory effects. Dysbiosis-induced impairment of gut barrier function and a reduction in beneficial microbial metabolites, such as SCFAs and sphingolipids, can compromise immune homeostasis (39, 93). For instance, a reduction in microbial sphingolipids due to cesarean section and decreased *Bacteroides* is implicated in increasing susceptibility to early asthma (93). Conversely, specific microbial metabolites are directly involved in disease pathways. Alterations in lipid metabolism, including elevations in ceramide species, are linked to allergen-induced apoptosis, oxidative stress, and neutrophilic inflammation in the lungs—hallmarks of severe asthma (94). Ceramide synthase 2 deficiency, which alters sphingolipid chain lengths, protects mice from allergic airway inflammation by modulating CD4^+^ T cell receptor signaling and Th2/Th17 responses (95). The fungal microbiome (mycobiome) also contributes to immune dysregulation. Indoor and airway mycobiomes differ between T2-high and T2-low asthma endotypes, with fungal community composition correlating with inflammatory markers like exhaled nitric oxide (96). Certain fungi, such as *Candida albicans*, can promote allergic airway responses through specific virulence factors like candidalysin, which stimulates platelets to release pro-inflammatory mediators (97).

**Figure 2:**
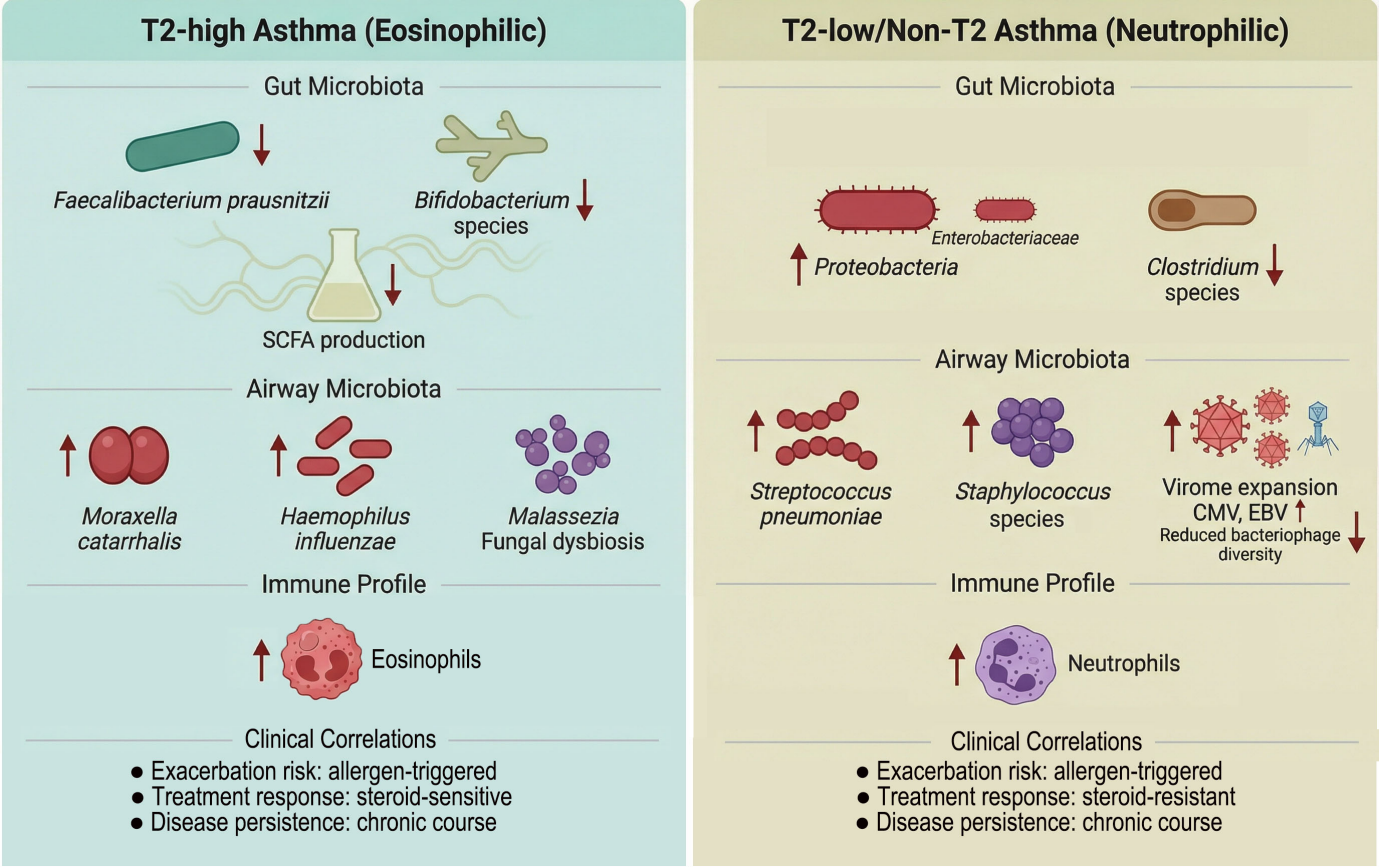
Distinct microbial signatures associated with childhood asthma endotypes. Comparative illustration of gut and airway microbiota profiles across different asthma endotypes. Left panel (T2-high asthma) is characterized by eosinophilic inflammation, with gut microbiota showing depletion of short-chain fatty acid-producing taxa (e.g., *Faecalibacterium prausnitzii*, *Bifidobacterium* species) and reduced SCFAs concentrations. Airway microbiota in T2-high asthma is often enriched for *Moraxella catarrhalis* and *Haemophilus influenzae*, with specific alterations in the mycobiome (e.g., *Malassezia* species). Right panel (T2-low/non-T2 asthma) features neutrophilic or paucigranulocytic inflammation, with gut microbiota exhibiting increased Proteobacteria (e.g., *Enterobacteriaceae*) and reduced Clostridia. Airway microbiota shows enrichment of *Streptococcus pneumoniae* and *Staphylococcus* species, accompanied by expansion of the eukaryotic virome (e.g., cytomegalovirus, Epstein-Barr virus) and reduced bacteriophage diversity. These microbial signatures correlate with clinical outcomes including exacerbation risk, treatment response, and disease persistence. ↑ indicates increased abundance; ↓ indicates decreased abundance. Microbial names are italicized following standard nomenclature. Abbreviations: CMV, cytomegalovirus; EBV, Epstein-Barr virus; SCFAs, short-chain fatty acids.

The development of this dysbiotic-immune axis is heavily influenced by environmental and host factors that converge during early life. A maternal high-fat diet can predispose offspring to severe lower respiratory infections and subsequent asthma by inducing neonatal low-grade systemic inflammation and gut dysbiosis, driven in part by neutrophil-mediated IL-6 trans-signaling (98). Early-life antibiotic exposure is a major disruptor, with different antibiotic classes promoting distinct types of skewed immune responses (Th2 or Th17) to allergens, linked to short- and long-term gut microbiota alterations and abnormal lipid metabolism in the lungs (99). The establishment of a diverse skin and mucosal microbiome, which is essential for seeding tissues with appropriate antigen-presenting cells, is itself dependent on the formation of a healthy microbiota in early life, creating a window of opportunity for preventing allergic inflammation (100). Importantly, the microbial and immune signatures of asthma are heterogeneous, reflecting the disease’s complex phenotypes. Integrated multi-omics studies reveal that specific endotypes can be identified even during infant bronchiolitis; for example, a cluster characterized by rhinovirus C infection, *Moraxella*-dominant microbiota, and a high T2 cytokine response carries a significantly elevated risk for developing childhood asthma (101). Similarly, in older patients, sputum microbial composition shows specific relationships with inflammatory phenotypes; a high Proteobacteria to Firmicutes ratio is associated with neutrophilic inflammation, whereas T2-high asthma may be associated with reduced bacterial burden in the airways, though this relationship can be complex and influenced by factors like fractional exhaled nitric oxide levels (102, 103). This heterogeneity underscores that the “microbial signature” of childhood asthma is not monolithic but a collection of distinct dysbiotic patterns associated with specific immune dysregulations, clinical presentations, and environmental histories, as contrasted for T2-high and T2-low endotypes in **Figure 2**.

## 5 Molecular mediators of cross-talk: microbial metabolites and immune modulation

**Figure 3:**
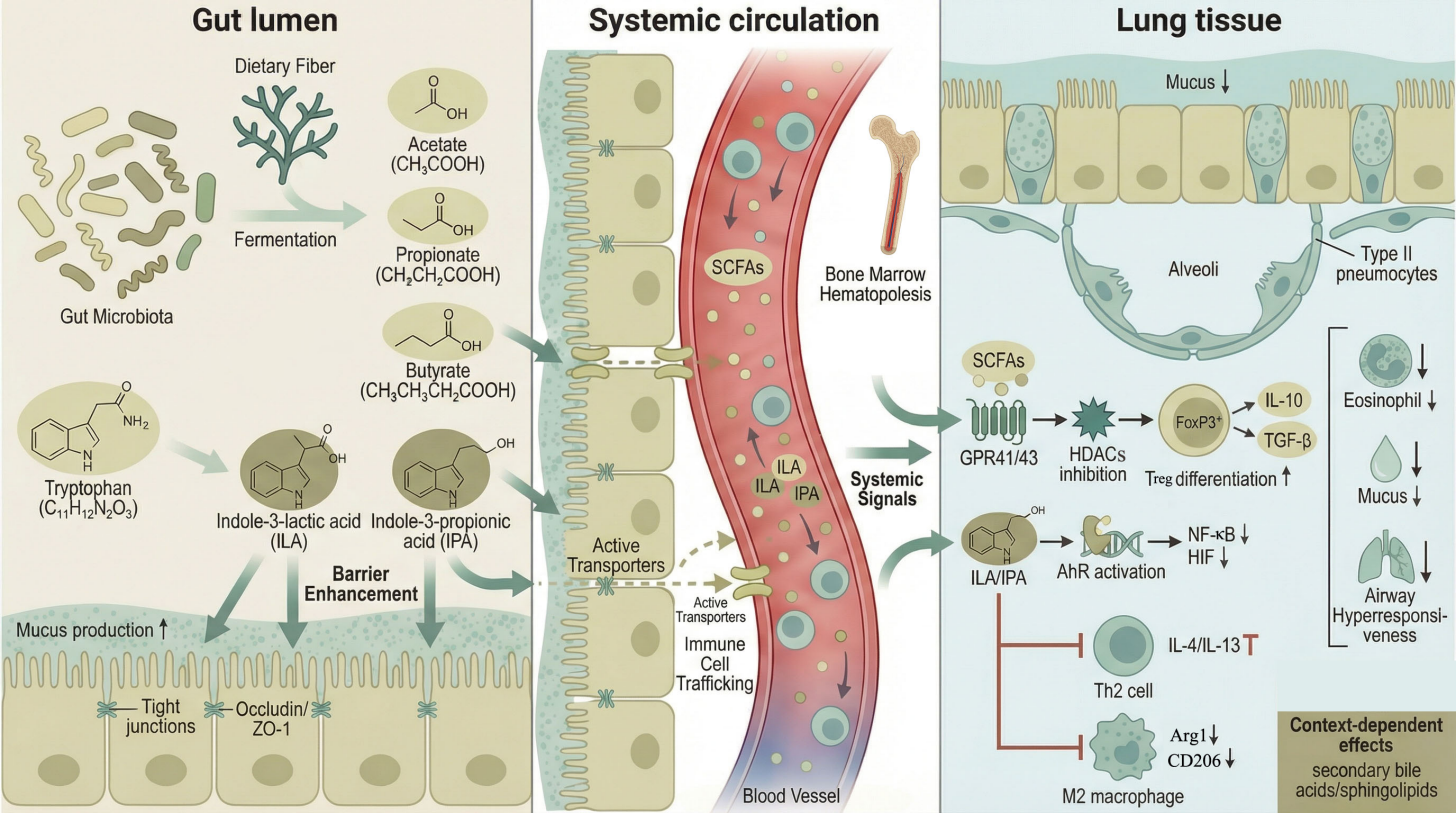
Molecular mechanisms of gut-lung axis crosstalk in childhood asthma. Schematic representation of the bidirectional communication between the intestinal microbiota and the pulmonary immune system, emphasizing the role of microbial metabolites as key molecular messengers. In the intestinal lumen (left panel), commensal bacteria ferment dietary fiber to produce short-chain fatty acids (SCFAs)—primarily acetate, propionate, and butyrate—and metabolize dietary tryptophan to indole derivatives such as indole-3-lactic acid (ILA) and indole-3-propionic acid (IPA). These metabolites enhance gut epithelial barrier integrity by promoting tight junction protein expression (occludin, ZO-1) and stimulating mucus production. Metabolites and microbial signals enter the systemic circulation (middle panel) via paracellular diffusion and active transport, where they influence immune cell trafficking and bone marrow hematopoiesis. In the lung tissue (right panel), SCFAs activate G protein-coupled receptors (GPR41/43) and inhibit histone deacetylases (HDACs), leading to epigenetic reprogramming that promotes regulatory T cells (Tregs) differentiation and suppressive function (FoxP3^+^, IL-10, TGF-β). SCFAs, particularly butyrate, also inhibit interleukin-4 (IL-4)-indu polarization (reduced Arg1, CD206 expression). Concurrently, tryptophan metabolites (ILA, IPA) activate the aryl hydrocarbon receptor (AhR) on epithelial and immune cells, downregulating pro-inflammatory pathways (NF-κB, HIF) and reinforcing barrier function. The collective effect is suppression of pathogenic T helper 2 (Th2) cell responses (IL-4, IL-13), reduced eosinophil recruitment, decreased mucus hypersecretion from goblet cells, and attenuated airway hyperresponsiveness. Lipid mediators, including secondary bile acids (e.g., isoDCA, deoxycholic acid) and sphingolipids (sphingosine-1-phosphate, ceramides), exert context-dependent immunomodulatory effects that further shape asthma susceptibility. Green arrows denote protective pathways promoting immune tolerance and barrier integrity; red T-bars indicate inhibitory effects on pro-inflammatory responses. Abbreviations: AhR, aryl hydrocarbon receptor; Arg1, arginase 1; CD206, cluster of differentiation 206; DCA, deoxycholic acid; FoxP3, forkhead box P3; GPR41/43, G protein-coupled receptor 41/43; HDACs, histone deacetylases; HIF, hypoxia-inducible factor; IL-4, interleukin-4; IL-10, interleukin-10; IL-13, interleukin-13; ILA, indole-3-lactic acid; IPA, indole-3-propionic acid; isoDCA, iso-deoxycholic acid; M2, alternatively activated macrophages; NF-κB, nuclear factor kappa-light-chain-enhancer of activated B cells; SCFAs, short-chain fatty acids; S1P, sphingosine-1-phosphate; TGF-β, transforming growth factor-beta; Th2, T helper 2 cell; Tregs, regulatory T cells ZO-1, zonula occludens-1.

The distinct dysbiotic patterns associated with childhood asthma exert their influence through a complex repertoire of microbially derived metabolites, which serve as key molecular messengers in the gut-lung axis. These metabolites, including SCFAs, tryptophan catabolites, bile acids, and various lipid mediators, modulate host immunity both locally and systemically, thereby shaping susceptibility to or protection from allergic airway inflammation. Among these, SCFAs—primarily acetate, propionate, and butyrate, produced by bacterial fermentation of dietary fiber—are the most extensively studied (104, 105). Their systemic immunomodulatory effects are mediated through multiple mechanisms: binding to G-protein-coupled receptors (GPCRs) such as GPR41 (FFAR3) and GPR43 (FFAR2), and inhibition of HDACs, leading to epigenetic reprogramming of immune cells (34, 104, 106). Butyrate, in particular, demonstrates potent protective effects in models of allergic asthma. It inhibits the IL-4-induced M2 polarization of alveolar macrophages, a process implicated in asthma pathogenesis, through mechanisms involving GPR43 activation and HDAC inhibition (107). Furthermore, butyrate enhances the integrity of mucosal barriers, not only in the gut but also in distal sites like the skin, by promoting keratinocyte metabolism and differentiation, thereby limiting systemic sensitization to allergens (108). This barrier-strengthening effect is complemented by the ability of SCFAs to promote the differentiation and suppressive function of Tregs, both in the periphery and in the intestine, which is crucial for maintaining immune tolerance (109, 110). Oral administration of specific probiotic strains, such as *Lactiplantibacillus plantarum* APsulloc331261, can ameliorate allergic airway inflammation and mucus hypersecretion by increasing gut-derived butyrate production (111). The production of SCFAs is developmentally regulated, with infant gut microbiota progressing through distinct metabolic phases characterized initially by low SCFAs and later by increased propionate and butyrate, a transition linked to breastfeeding cessation and the expansion of Clostridiales species possessing the acetyl-CoA pathway (55). Consequently, interventions that boost SCFAs levels, such as synbiotic mixtures or fecal microbiota transplantation (FMT), have shown promise in improving lung health and attenuating airway inflammation in preclinical models, often correlating with increased SCFAs concentrations in the gut (112, 113). The complex network of molecular signals mediating gut-lung crosstalk, from microbial metabolite production to systemic immune modulation and pulmonary effects, is summarized in **Figure 3**.

Beyond SCFAs, microbiota-derived tryptophan metabolites constitute another critical class of immunomodulators. Bifidobacteria species prevalent in breastfed infants metabolize aromatic amino acids into aromatic lactic acids, such as indole-3-lactic acid (ILA) (114). ILA and related metabolites like indole-3-carboxaldehyde (I3C) and indole-3-propionic acid (IPA) exert anti-inflammatory effects primarily by acting as agonists for AhR, a ligand-activated transcription factor abundantly expressed at mucosal surfaces (114–116). Activation of AhR by these metabolites downregulates pro-inflammatory pathways, including NF-κB and HIF signaling, in intestinal epithelial cells, thereby reducing the production of chemokines like CCL2/7 that recruit inflammatory macrophages (115, 116). In the context of asthma, supplementation with the probiotic *Bifidobacterium animalis* subsp. *lactis* CCFM1274, which remodels intestinal tryptophan metabolism, alleviated allergic asthma symptoms in mice (117). Crucially, early-life antibiotic-driven dysbiosis leads to a long-term reduction in systemic IPA levels, which alters lung epithelial mitochondrial metabolism and stress responses, predisposing to exacerbated allergic airway inflammation in adulthood; this predisposition can be prevented by early-life IPA supplementation (118). Tryptophan metabolites also contribute to the efficacy of allergen-specific immunotherapy by promoting Tregs responses and attenuating airway inflammation (119). Another tryptophan-derived molecule, gut bacteria-produced serotonin, is enriched in the neonatal gut and promotes the differentiation of peripheral Tregs, establishing long-term antigen-specific immune tolerance that is protective against allergic inflammation (120).

Lipid mediators of microbial origin, including bile acids and sphingolipids, further diversify the metabolic cross-talk influencing asthma. Gut bacteria transform primary bile acids into secondary bile acids, such as isoDCA and deoxycholic acid (DCA), which can regulate immune responses. IsoDCA promotes the differentiation of colonic Tregs by modulating the immunostimulatory properties of dendritic cells via the farnesoid X receptor (FXR) (109). Conversely, certain dietary fibers like inulin can, in a microbiota-dependent manner, elevate specific bile acids like cholic acid, which subsequently promote T2 inflammation at barrier surfaces through FXR signaling, potentially exacerbating allergic responses (121). This highlights the context-dependent and sometimes opposing roles of microbial metabolites. Sphingolipid metabolites are also implicated in asthma pathophysiology. Sphingosine-1-phosphate (S1P) can enhance airway smooth muscle hyperresponsiveness and proliferation, key features of asthma, via the S1P receptor type 2 (122). Ceramides, another class of sphingolipids, are elevated in the lungs after allergen challenge and contribute to apoptosis, oxidative stress, and neutrophilic inflammation, hallmarks of severe asthma (94). Genetic ablation of ceramide synthase 2, which produces very-long-chain ceramides, protects mice from allergic airway inflammation by altering CD4^+^ T cell responses, indicating the chain-length-specific role of sphingolipids in immune regulation (95). Other bacterial fatty acid metabolites, such as the long-chain fatty acid 3-hydroxyoctadecaenoic acid (C18-3OH) produced by *Escherichia coli* Nissle 1917 and *Holdemanella biformis*, exhibit anti-inflammatory properties by acting as agonists for peroxisome proliferator-activated receptor gamma (PPARγ) (123, 124).

The collective action of these metabolites creates a complex network that influences the host immune system’s set point. They regulate the balance between pro-inflammatory and tolerogenic responses across multiple cell types: promoting Treg and anti-inflammatory macrophage differentiation, strengthening epithelial barriers, inhibiting pathogenic Th2 and Th17 responses, and modulating B cell antibody class-switching through epigenetic mechanisms (107, 110, 125, 126). However, the net immunological outcome is highly dependent on the metabolite’s concentration, the host’s developmental stage, genetic background, and the composition of the broader microbial community. For instance, while SCFAs generally promote tolerance, their effects on B cells can be bidirectional, enhancing or inhibiting antibody responses based on concentration (126). Similarly, the impact of bile acids can shift from anti-inflammatory to pro-inflammatory depending on the dietary context and bacterial transformations (109, 121). This complexity is reflected in human studies, where altered host immunoglobulin A (IgA) responses to specific gut bacteria, rather than mere changes in microbiota composition, are associated with asthma severity, suggesting that the host’s immune recognition and handling of microbial antigens and metabolites are critical (127). The translation of this knowledge into therapies is an active area of investigation, focusing on dietary interventions, prebiotics, probiotics, synbiotics, and postbiotics (defined microbial metabolites) to favorably modulate this metabolic dialogue for asthma prevention and management (128, 129).

## 6 Environmental and host factors shaping the microbiome and asthma risk

The intricate metabolic dialogue between the gut microbiome and the host immune system does not occur in a vacuum. It is profoundly shaped by a constellation of environmental exposures and host-specific factors acting from the prenatal period throughout early childhood. These influences collectively determine the trajectory of microbial community assembly, its functional maturation, and ultimately, the immunological set point that confers resilience or susceptibility to asthma (53, 54). The prenatal period represents a foundational stage. Maternal diet during pregnancy directly programs the infant’s early microbial and immune landscape. A maternal high-fat diet has been shown to induce neonatal low-grade systemic inflammation and gut dysbiosis, predisposing offspring to severe lower respiratory tract infections and subsequent asthma via neutrophil-mediated IL-6 trans-signaling (98). Conversely, prenatal supplementation with n-3 long-chain polyunsaturated fatty acids (fish oil) or high-dose vitamin D has been associated with a reduced risk of early childhood croup, suggesting an early immunomodulatory benefit (130). Antibiotic use during pregnancy is a significant risk factor, linked to offspring gut dysbiosis, intestinal barrier disruption, and increased asthma severity in a dose-dependent manner in experimental models (46, 47). This effect may synergize with delivery mode; children born by cesarean section and exposed to prenatal antibiotics exhibit the highest asthma risk, potentially through synergistic alterations in early gut microbiota (131). The mode of delivery itself is a major determinant of initial gut colonization. While cesarean section causes marked early microbial perturbations, the persistence of this “cesarean signature” at one year of age, rather than the delivery mode itself, is associated with increased asthma risk, underscoring the importance of postnatal microbiome maturation in mitigating initial disruptions (51).

Postnatal environmental exposures continue to sculpt the microbiome during the critical window of immune development. The protective “farm effect” against asthma is a paradigmatic example of beneficial environmental programming. This protection is mediated, in part, by enhanced gut microbiome maturation and increased production of microbial metabolites like butyrate (53). Continuous exposure, such as the sustained consumption of farm milk from infancy through school age, appears necessary for lasting protection against conditions like hay fever, arguing against the notion that only very early exposures set irreversible trajectories (132, 133). In contrast, urbanization is associated with distinct, less protective microbiota profiles in both the airway and gut of infants, which correlate with an increased risk of asthma and atopy, likely by altering cross-talk with the developing immune system (134). Sibling exposure, particularly a smaller age gap to the closest older sibling, is another powerful determinant of infant microbial composition in the airway and gut, and this microbial signature is associated with protection against asthma (59). Similarly, prenatal dog keeping enriches infant gut microbial diversity and specific taxa like *Fusobacterium*, supporting the microbial hypothesis underlying the observed reduction in allergy and asthma risk associated with pet exposure (62). The indoor environment itself is a source of microbial and metabolic exposures; indoor microbial communities and metabolites, such as indole derivatives, have been linked to the composition and health index of children’s gut microbiota, suggesting a direct role of the home ecosystem in shaping resident microbiomes (135).

Host genetics constitute a key layer that interacts with these environmental determinants. Large-scale genome-wide association studies have identified host genetic loci that influence gut microbiome composition (136). More specifically, a polygenic risk score for asthma can modulate the effect of specific gut bacteria on respiratory outcomes; for instance, protective associations of species like *Ruminococcus bromii* with asthma were more pronounced in children with a high genetic risk (5). Furthermore, the 17q12–21 asthma risk genotype and gut microbial maturation exert independent and additive effects on childhood asthma risk, indicating that genetic susceptibility and microbiome-mediated environmental influences operate through at least partially distinct pathways (54). Racial disparities in asthma prevalence may also be partially rooted in microbiome differences. In children with food allergy, Black children exhibited a gut microbiota composition distinct from White children, characterized by differences in *Bacteroides* and *Bifidobacterium* species, and these variations were associated with a history of asthma (88).

Dietary patterns across the lifespan are fundamental modulators of the gut ecosystem. Breastfeeding influences asthma risk, an association mediated in part by its effect on early gut microbial maturation (54). For non-breastfed infants, especially those with cow’s milk protein allergy, formulas supplemented with HMOs support normal growth and may reduce the risk of respiratory infections (137). During the complementary feeding period and beyond, dietary fiber intake is crucial. High-fiber diets are associated with a lower risk of asthma, wheeze, and systemic inflammation in adults, likely through the promotion of a SCFAs-producing microbiota and downstream immune regulation (138, 139). The Mediterranean diet, rich in fiber and polyphenols, is likewise linked to beneficial gut microbiome profiles (140). In contrast, early-life antibiotic exposure, a major disruptor of microbial communities, remains one of the most consistent risk factors for childhood asthma, with population-level decreases in antibiotic prescribing correlating with reduced asthma incidence (56, 58). The impact varies by antibiotic class and dose, and can have long-term effects on gut microbial composition (99, 141).

Beyond the bacterial microbiome, other microbial kingdoms and body sites contribute to the network of exposures. The gut mycobiota (fungal community) undergoes dramatic shifts in the first year of life, is shaped by environmental factors like geography and diet, and its composition can predict subsequent inhalant atopy (142). The oral and gut microbiomes are interconnected, with oral bacteria capable of ectopic gut colonization, a process observed in conditions like alcohol dependence (143). Periodontopathic bacteria can translocate to the placenta, potentially influencing the prenatal inflammatory milieu (144). Furthermore, the lung mycobiome shows substantial overlap with the fungal community of the domestic environment, indicating direct environmental seeding of respiratory microbial niches (145).

These myriad factors interact in complex ways. Host genetics can modify the impact of environmental microbial exposures (5). The effect of prenatal exposures can be amplified or mitigated by postnatal events (131). The protective farm effect requires sustained exposure (132). This intricate interplay underscores that childhood asthma risk emerges from the dynamic convergence of genetic predisposition and a lifetime of environmental exposures that collectively program the gut-lung axis. Mendelian randomization studies support a genetically predicted causal role for specific gut microbial genera in asthma and its phenotypes, highlighting taxa like *Holdemanella*, *Ruminococcaceae UCG014*, and *Oxalobacter* as potential players (146–148). However, the bidirectional nature of host-microbe interactions and the challenge of accounting for all confounders mean that establishing definitive causality in humans remains complex (149). Understanding these multifactorial relationships is essential for developing targeted strategies to promote a resilient, asthma-protective microbiome from the earliest stages of life.

## 7 Microbiome-targeted interventions: from mechanisms to therapeutic potential

**Figure 4:**
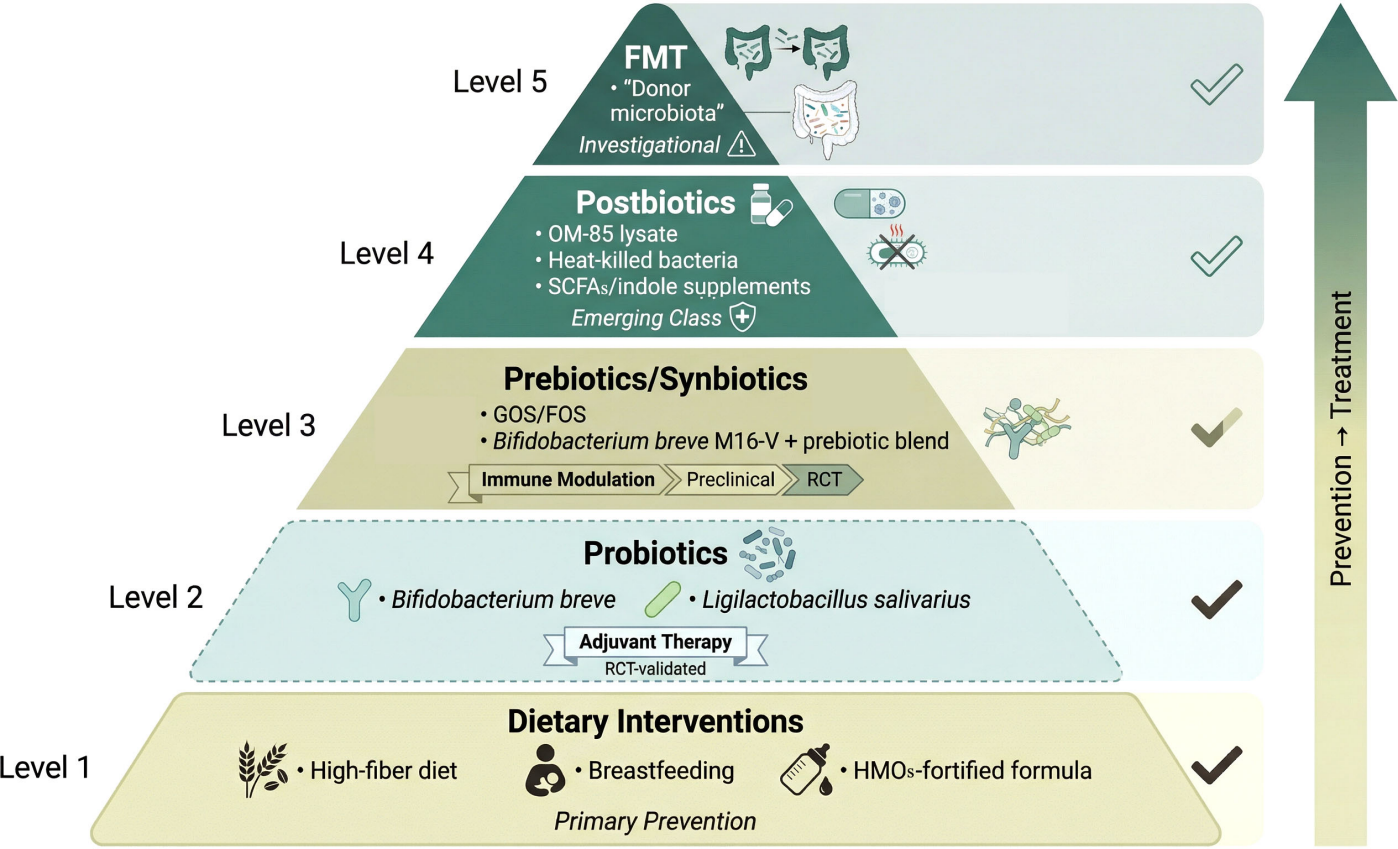
Microbiome-targeted interventions for childhood asthma: current evidence and future directions. Pyramid diagram depicting the spectrum of microbiome-directed therapeutic strategies, organized by level of intervention intensity and evidence maturity. Level 1 (base): Dietary interventions including high-fiber intake, breastfeeding, and human milk oligosaccharides (HMOs)-fortified formulas, supported by observational and mechanistic studies for primary prevention. Level 2: Probiotic supplementation with strain-specific formulations (e.g., *Bifidobacterium breve*, *Ligilactobacillus salivarius*), supported by randomized controlled trials (RCT) demonstrating reduction in exacerbation frequency. Level 3: Prebiotics and synbiotics (e.g., GOS/FOS, *Bifidobacterium breve* M16-V with prebiotic blends) with preclinical and early clinical evidence for immune modulation. Level 4: Postbiotics including bacterial lysates (OM-85), heat-killed bacteria, and defined microbial metabolites (SCFAs, indole derivatives), representing an emerging class with favorable safety and stability profiles. Level 5 (apex): Fecal microbiota transplantation (FMT) as a whole-ecosystem restoration approach, currently investigational for severe or refractory asthma with evidence limited to preclinical models and case reports. The right vertical arrow indicates the spectrum from prevention-oriented strategies (base) toward therapeutic interventions for established disease (apex). Abbreviations: FMT, fecal microbiota transplantation; GOS, galacto-oligosaccharides; FOS, fructo-oligosaccharides; RCT, randomized controlled trials; HMOs, human milk oligosaccharides; OM-85, bacterial lysate; SCFAs, short-chain fatty acids.

Given the established link between early-life dysbiosis, immune dysregulation, and subsequent asthma development, strategies aimed at restoring or promoting a healthy microbiome have emerged as promising therapeutic avenues. These interventions, targeting the gut-lung axis, range from direct microbial supplementation and dietary modulation to more radical approaches like FMT, each with distinct mechanisms and stages of clinical validation. A systematic review evaluating probiotics, prebiotics, synbiotics, and postbiotics in pediatric asthma concluded that these microbiota-targeted therapies show potential, particularly in reducing asthma exacerbations and improving pulmonary function, but also highlighted significant heterogeneity in study designs and a need for standardized protocols (150). These strategies, ranging from dietary modulation to FMT, are hierarchically organized in **Figure 4** according to intervention intensity and evidence maturity.

Probiotics, defined as live microorganisms that confer a health benefit when administered in adequate amounts, represent the most extensively studied intervention. Clinical trials in children with asthma have demonstrated that specific probiotic strains or mixtures can reduce the frequency of exacerbations, improve asthma control scores, and modulate immune parameters. The PROPAM study, a randomized controlled trial in a primary care setting, showed that a mixture of *Ligilactobacillus salivarius* LS01 and *Bifidobacterium breve* B632 significantly reduced the number of asthmatic exacerbations in children (151). Another RCT found that adjunctive administration of *Bifidobacterium lactis* Probio-M8 alongside conventional therapy improved asthma control and reduced airway inflammation markers, an effect associated with increased gut microbiome resilience and changes in serum and microbial metabolites (152). These benefits are often linked to the modulation of gut microbiota composition, such as increasing the abundance of beneficial genera like *Faecalibacterium* and *Veillonella* while decreasing *Bacteroides*, and to the correction of immune imbalances, including a reduction in serum IgE and Th2 cytokines like IL-4 and IL-13 (153). However, these findings must be interpreted with caution due to several important limitations. The PROPAM study had a relatively modest sample size and was conducted in a single primary care setting, potentially limiting generalizability and introducing selection bias (151). More broadly, a recent systematic review highlighted significant heterogeneity across study designs, interventions, and outcome measures in probiotic trials for pediatric asthma, precluding definitive conclusions and emphasizing the need for standardized protocols and larger, well-powered randomized controlled trials (150). Furthermore, publication bias—the tendency for positive results to be more frequently published—is well-documented in the probiotic literature and may lead to overestimation of treatment effects. Collectively, these considerations underscore that the current evidence for probiotic efficacy in pediatric asthma remains preliminary and mixed. The effects of probiotics are highly strain-specific and dependent on viability status. Different strains of the same species, such as various *Lactobacillus casei* strains, can exert differential prophylactic effects on house dust mite-induced asthma in mice by uniquely affecting immune responses, gut microbiota composition, and SCFAs production (154). Similarly, the allergy-protective effects of *Bifidobacterium longum* were shown to be significantly attenuated, though not completely abolished, when the bacteria were heat-inactivated, underscoring that viability can be a prerequisite for optimal benefit (155). This strain-specificity underscores the necessity for target-based probiotic development using host-adapted strains with known molecular effectors (156).

Beyond probiotics, postbiotics—defined as “preparations of inanimate microorganisms and/or their components that confers a health benefit on the host”—offer a promising alternative with advantages in safety, stability, and storage (157). These include bacterial lysates, heat-killed bacteria, and purified microbial components. Postbiotics such as the bacterial lysate OM-85 have been shown in clinical studies to attenuate airway hyperresponsiveness and systemic inflammation in pediatric asthma (150). Mechanistically, postbiotics can exert immunomodulatory effects; for example, lysates from *Bacillus velezensis* Kh2–2 enhanced immune responses via NF-κB and MAPK signaling pathways (158). In animal models, aerosol inhalation of heat-killed *Clostridium butyricum* effectively alleviated allergic airway inflammation by restoring Th1/Th2 balance and inhibiting the NF-κB/NLRP3 inflammasome pathway, demonstrating a route of administration that directly targets the lung (159). The role of postbiotics in asthma treatment is gaining recognition for their potential to lower exacerbation frequency by modulating the host immune response (160).

Synbiotics, which combine probiotics with prebiotics (selectively fermented ingredients that allow specific changes in the gut microbiota), aim to synergistically enhance the survival and activity of beneficial microbes. A synbiotic mixture of *Bifidobacterium breve* M16-V and a prebiotic fiber blend (scGOS/lcFOS/pectin) protected against lung function decline and pulmonary neutrophilia in a murine model, effects that correlated with enhanced fecal SCFAs production, suggesting a gut-lung axis-mediated mechanism (112). Clinically, synbiotic interventions have been associated with a reduction in viral respiratory infections and healthcare utilization in children, which is particularly relevant as respiratory infections are common triggers for asthma exacerbations (150).

In addition to direct microbial supplementation, dietary interventions that shape the gut microbiota ecosystem are a fundamental preventive and therapeutic strategy. Prebiotic fibers are fermented by gut bacteria to produce SCFAs, which have well-established anti-inflammatory and immune-regulatory properties. While evidence directly linking prebiotic supplementation to improved asthma outcomes in children is still limited (150), their role in promoting immune health is supported by their effects on microbial metabolism and immune regulation (139). HMOs, natural prebiotics in breast milk, when added to infant formula, have shown a protective effect against respiratory and ear infections in the first year of life (137). However, a large RCT found that maternal supplementation with GOS/FOS during pregnancy and lactation did not reduce the risk of medically diagnosed eczema by age one in infants with a hereditary risk of allergy, highlighting the complexity of timing and population selection in preventive nutritional strategies (161). Other dietary components like the polyphenol resveratrol have been shown in murine studies to attenuate allergic asthma by enriching beneficial microbiota and increasing butyrate production, thereby strengthening the epithelial barrier and reducing inflammation (129).

For more severe dysbiosis or as an investigational therapy, FMT represents a “whole microbiome” replacement strategy. In preclinical models, FMT from healthy donors to asthmatic rats alleviated airway inflammation and increased intestinal SCFAs levels (113). Furthermore, restoring gut microbiota via FMT in ovalbumin-challenged mice partially corrected respiratory microbiota dysbiosis and significantly alleviated airway inflammation, providing direct experimental evidence for the therapeutic potential of modulating the gut-lung axis (162). Despite these promising preclinical findings, the translation of FMT to pediatric asthma faces substantial safety and regulatory hurdles that warrant careful consideration. First, FMT carries inherent risks of pathogen transmission; despite rigorous donor screening, cases of extended-spectrum beta-lactamase (ESBL)-producing *Escherichia coli* bacteremia and other serious adverse events have been reported, prompting the U.S. Food and Drug Administration (FDA) to issue multiple safety alerts, including warnings regarding transmission of enteropathogenic *E. coli* and Shiga toxin-producing *E. coli*, and fatality due to multidrug-resistant organism transmission (163). Second, the long-term consequences of introducing a foreign microbial ecosystem into a developing pediatric host remain unknown, raising concerns about microbiome stability and potential unintended effects on immune maturation, metabolic programming, and neurodevelopment. Third, regulatory frameworks for FMT in non-CDI indications remain nascent; in the United States, FMT for indications other than recurrent C. difficile infection requires an Investigational New Drug (IND) application, adding complexity to clinical translation. Fourth, pediatric applications pose unique challenges, including the need for age-matched donors, consideration of developmental stage-specific microbial requirements, and ethical considerations surrounding informed consent in minors. These safety concerns are particularly salient in asthma, a non-life-threatening condition where the risk-benefit ratio must be carefully evaluated before considering FMT as a therapeutic option. Consequently, while FMT offers valuable mechanistic insights in preclinical models, its clinical application in pediatric asthma remains investigational and should be pursued only within the context of rigorously designed clinical trials with comprehensive safety monitoring (163).

The timing of intervention is a critical factor. The perinatal period and early infancy represent a key window for microbiome “programming” with long-term health consequences (164). While some studies on prenatal probiotic administration have shown inconsistent results in preventing allergies and asthma in offspring, there is indication that benefits may be more pronounced in high-risk groups (165). A narrative review concluded that there is currently little evidence to recommend pro-, pre-, or synbiotics for the *prevention* of asthma and allergic rhinitis in children, largely due to confounding factors like environment and strain selection (166). This contrasts with more promising data for therapeutic use in already established disease (150, 151). Therefore, interventions may need to be tailored not only to the individual’s microbial and immune phenotype but also to the specific life stage.

## 8 Heterogeneity and future directions: towards microbiome-informed precision medicine in asthma

The profound heterogeneity of asthma, encompassing diverse clinical phenotypes and molecular endotypes, presents a major challenge for effective management and underscores the necessity for precision medicine approaches (167–170). This heterogeneity is mirrored and potentially driven by significant variations in microbial ecology across body sites and individuals (21, 171). In the context of early-life programming and childhood asthma pathogenesis, the predominant direction of evidence supports gut-to-lung signaling, whereby intestinal dysbiosis and microbial metabolite alterations shape pulmonary immune development and allergic susceptibility. However, emerging evidence also supports the reverse direction: respiratory infections and inflammation can reciprocally influence gut homeostasis. For example, respiratory syncytial virus (RSV)-induced lung inflammation has been shown to increase intestinal permeability through systemic cytokine spillover, and severe asthma exacerbations have been associated with alterations in gut microbiota composition, suggesting that pulmonary inflammation may feed back to disrupt gut microbial ecosystems (76, 77). Nevertheless, mechanistic evidence for lung-to-gut signaling in the specific context of childhood asthma programming remains limited and warrants further investigation. The respiratory microbiome itself is not a monolith; distinct communities exist in the upper versus lower airways, and each compartment—bacterial, fungal, and viral—contributes uniquely to disease pathogenesis (82, 84, 96, 172). The nasal microbiome during respiratory health and illness exhibits seasonal dynamics and harbors specific bacterial networks whose interaction with host epithelial transcriptional modules significantly increases exacerbation risk in a season-specific manner (81). Simultaneously, the gut microbiome demonstrates phenotype-specific signatures, with systems-level alterations in microbial carbohydrate-active enzymes and virulomes distinguishing children with asthma from those with allergic rhinitis or healthy controls (173). This complex mosaic necessitates a shift from viewing the microbiome as a singular entity to deciphering how specific, context-dependent host-microbe interactions underpin discrete asthma subgroups.

Crucially, microbial signatures are intrinsically linked to established asthma endotypes and clinical presentations (80, 167). T2-high asthma is associated with a specific indoor mycobiome and microbiome profile, including decreased fungal alpha diversity indoors, which correlates with elevated fractional exhaled nitric oxide (FeNO) (96). In contrast, T2-low or non-T2 inflammation is often associated with neutrophilic pathways and distinct microbial profiles, such as those high in Proteobacteria, which relate to longer asthma duration and are not effectively identified by eosinophilic biomarkers alone (103, 168). Integrative multi-omic studies are pivotal for unraveling these connections. Unsupervised multiomic factor analysis has delineated a factor for recurrent preschool wheeze driven by type 1 immunity and neutrophil-associated lipids, distinct from a factor capturing progression to school-aged asthma dominated by epithelial gene expression, T2 responses, and increased *Haemophilus influenzae* (174). Similarly, integrated-omics endotyping of infants with rhinovirus bronchiolitis identified a high-risk subgroup characterized by RV-C infection, *Moraxella*-dominant microbiota, and a high T2 cytokine response, which was significantly associated with later childhood asthma development (101). These studies highlight how integrating microbial data with host transcriptomic, metabolomic, and clinical data can reveal biologically meaningful, stable subgroups (170).

Future progress hinges on employing systems biology and advanced integrative analytics to model these complex, multi-kingdom interactions (170, 175). Approaches such as latent Dirichlet allocation (LDA) can connect specific microbes to host gene expression patterns in heterogeneous sputum samples, uncovering novel relationships in asthma (176). Investigating the cross-kingdom ecological network reveals a dense and homogenous correlation structure in health that becomes dramatically unbalanced in asthma, indicating disease-specific alterations in fungal-bacterial interactions (177). Furthermore, moving beyond simple abundance metrics to understand functional guilds within the microbiome, such as the “two competing guilds” model of fiber-fermenting butyrate producers versus virulence-associated taxa, offers a holistic framework for identifying a core health-relevant microbiome signature (178). This guild-based, interaction-focused approach could serve as a common target for health enhancement. The goal is to define these functional networks and their key mediators—such as SCFAs or specific immunomodulatory metabolites—to identify actionable therapeutic targets within the dysbiotic ecosystem (179).

A primary translational application of microbiome research is the development of predictive biomarkers for disease trajectory and treatment response (82, 180). The upper airway mycobiome, particularly the relative abundance of commensal *Malassezia globosa*, is associated with a lower risk of future loss of asthma control and progression to severe exacerbation (82). Specific nasal microbiota configurations during respiratory illnesses can predict exacerbation progression (81). These findings suggest that microbial profiling could stratify patients based on exacerbation risk. However, significant challenges remain in translating these associations into clinically actionable, real-time prediction tools (180). Future models will likely need to incorporate dynamic, multi-omic data with clinical variables to achieve reliable prediction of impending exacerbations and identify responders to conventional or novel microbiome-directed therapies (103, 180).

**Figure 5:**
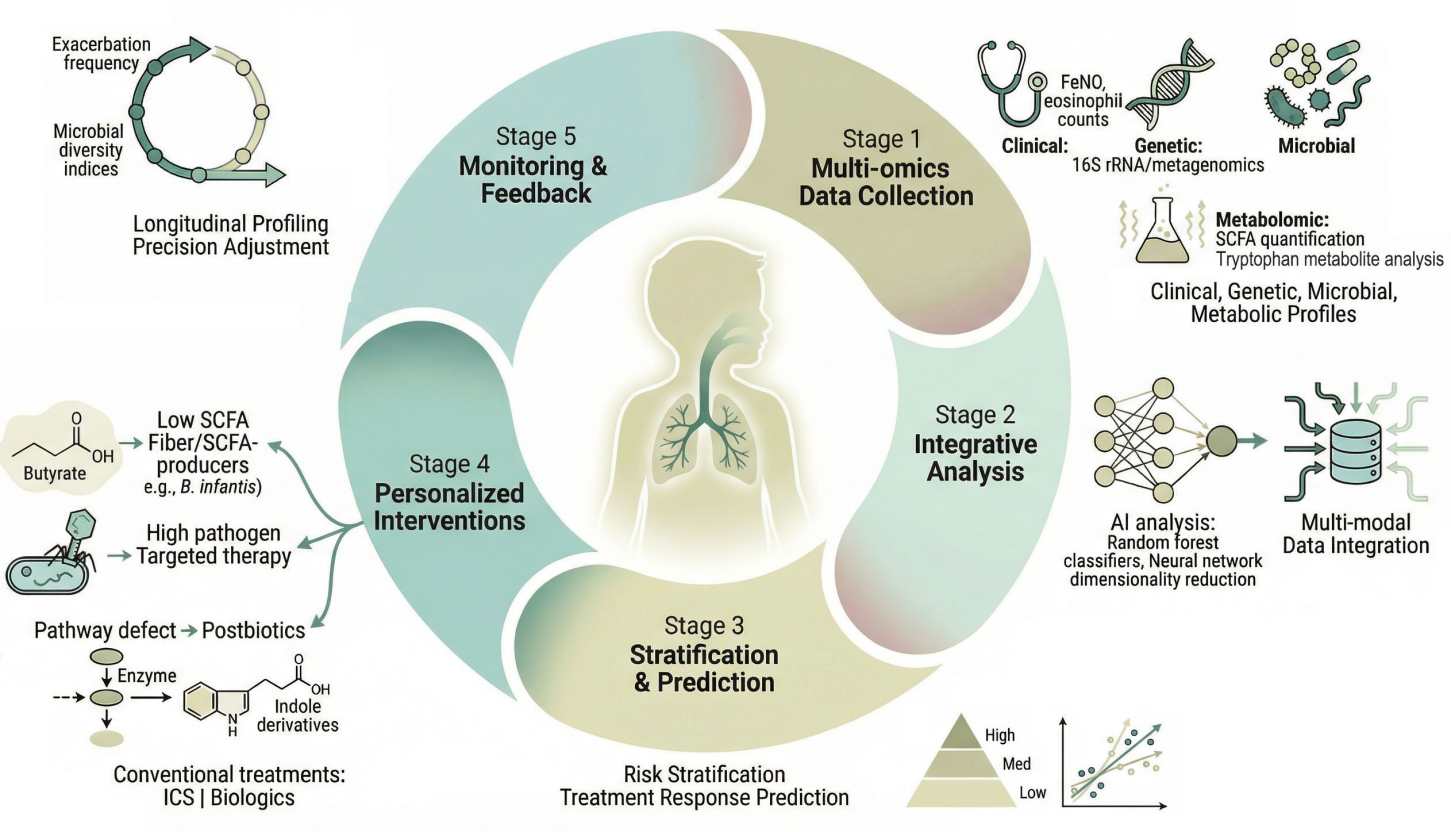
A Precision medicine framework integrating the gut-lung axis in childhood asthma. Proposed clinical algorithm for microbiome-informed precision management of childhood asthma. The cycle begins with (1) multi-omics data acquisition, encompassing clinical phenotyping (symptoms, exacerbation history), inflammatory biomarkers (fractional exhaled nitric oxide [FeNO], blood and sputum eosinophils), host genetics (polygenic risk scores), multi-kingdom microbial profiling (16S rRNA sequencing or metagenomics of gut and airway samples), and metabolomics (quantification of short-chain fatty acids [SCFAs] and tryptophan-derived metabolites such as indole-3-lactic acid [ILA] and indole-3-propionic acid [IPA]). These data are integrated through (2) advanced computational analysis employing machine learning algorithms to identify stable endotypes and predict individual disease trajectories. (3) Stratification and prediction enable classification into risk categories and prediction of treatment response to both conventional therapies (inhaled corticosteroids, biologics) and microbiome-directed interventions. (4) Personalized intervention selection matches patients to targeted strategies based on their specific microbial-metabolic deficits: dietary fiber or SCFAs-producing probiotics (e.g., *Bifidobacterium infantis*) for “low-SCFAs” profiles; targeted antimicrobial or competitive exclusion strategies for “high-pathogen” profiles; and postbiotic supplementation for specific pathway defects (e.g., indole-3-propionic acid deficiency). (5) Longitudinal monitoring and feedback allows dynamic treatment adjustment based on clinical response and repeated microbial-metabolic profiling, completing the cycle. This framework represents a paradigm shift from one-size-fits-all management toward personalized, mechanism-based care targeting the gut-lung axis. Abbreviations: FeNO, fractional exhaled nitric oxide; ILA, indole-3-lactic acid; IPA, indole-3-propionic acid; SCFAs, short-chain fatty acids; ICS, inhaled corticosteroids.

Ultimately, the path towards microbiome-informed precision medicine involves integrating microbial signatures into a multidimensional stratification framework to guide personalized intervention (80, 181). Microbial community data can help delineate asthma phenotypes, as demonstrated by clusters driven by genera like *Haemophilus* and *Neisseria*, which are associated with fixed airflow obstruction and mixed granulocytic inflammation in children (80). Gut microbiome profiles also differ significantly between allergic and non-allergic asthma endotypes, with specific bacterial species and functional pathways showing strong diagnostic potential (182). This stratification can inform therapeutic choices; for instance, high FeNO may identify a T2-high subgroup with a less Proteobacteria-dominated microbiome, potentially less likely to benefit from broad antimicrobial strategies, whereas those with low FeNO and a dysbiotic, high-Proteobacteria profile might be candidates for microbiome-modulating approaches (103). The future therapeutic arsenal may therefore include not only biologics targeting specific immune pathways but also next-generation probiotics, prebiotics, postbiotics, or even microbiota transplantation, selected based on an individual’s specific microbial and immunologic deficit (21, 181). Success in this endeavor depends on rigorous validation in large-scale, longitudinal cohorts and clinical trials that treat the microbiome as a modifiable component of the host-environment interface central to asthma pathogenesis and prevention (71, 167). It is important, however, to acknowledge the current translational gap between this aspirational framework and routine clinical practice. Multi-kingdom microbial profiling and comprehensive metabolomics remain research tools not yet routinely available or reimbursed in clinical settings. Currently actionable strategies include promoting protective factors (breastfeeding, judicious antibiotic use, pet exposure) and recognizing that while microbial signatures correlate with asthma endotypes, they are not yet ready for clinical decision-making. Comprehensive multi-omics profiling, strain-specific probiotics, and FMT for asthma remain investigational and should be pursued only within research contexts. Ultimately, the path towards microbiome-informed precision medicine involves integrating microbial signatures into a multidimensional stratification framework to guide personalized intervention, as proposed in **Figure 5**. This cyclical algorithm encompasses multi-omics data acquisition, integrative analysis, risk stratification, personalized intervention selection, and longitudinal monitoring, representing a paradigm shift toward mechanism-based care targeting the gut-lung axis.

## 9 Conclusion and future perspectives

In conclusion, the cumulative evidence firmly establishes the gut-lung axis as a cornerstone in the developmental programming and pathophysiology of childhood asthma. The early-life period represents a critical window where environmental exposures, through their impact on gut and airway microbial colonization and diversity, imprint lasting effects on immune maturation and disease susceptibility (1, 78). Dysbiosis, characterized by reduced overall diversity and altered abundances of specific bacterial taxa in both the gut and lung, is intricately linked to the risk and phenotypic manifestations of asthma (16, 21, 79). This dysbiosis disrupts the homeostatic crosstalk along the gut-lung axis, leading to dysregulated immune responses—often skewed towards a T2 inflammatory profile (2, 175). Microbial metabolites, particularly SCFAs, serve as crucial molecular messengers in this axis, translating microbial signals into systemic and local immunomodulatory effects (16, 19, 183). While these mechanistic insights are compelling, the translation of this knowledge into effective, microbiome-informed clinical strategies for asthma prevention and management remains a work in progress, highlighting several key areas for future research and application.

A primary challenge lies in harmonizing the often heterogeneous and sometimes inconsistent findings from human cohort studies, as noted in systematic reviews (184). Future efforts must prioritize large-scale, longitudinal birth cohorts that employ deep, multi-omic profiling—integrating metagenomics, metabolomics, and host transcriptomics—to move beyond associative links and elucidate causal pathways (174, 175). Such studies, utilizing shotgun metagenomic sequencing from the first weeks of life, are essential for resolving microbial communities to the species or strain level and linking specific microbial functions to asthma endotypes (174, 184). This granular understanding is a prerequisite for developing reliable microbial biomarkers for risk stratification. Furthermore, a holistic life-course perspective that integrates the maternal prenatal microbiome, the infant’s early-life gut and respiratory microbiomes, and their dynamic interactions with host immunity will provide a more comprehensive framework for understanding asthma origins (1, 43).

The ultimate goal is to leverage this knowledge for targeted interventions. Current approaches such as probiotic supplementation show promise but have yielded mixed results, underscoring the need for more personalized, condition-specific formulations (16, 79). Future therapeutic innovation will likely move towards next-generation, rationally designed microbial consortia, precision prebiotics, and defined postbiotic metabolites (e.g., SCFAs) tailored to correct individual-specific microbial deficits (16, 18, 183). The potential of FMT as a more radical ecosystem-restoring therapy for severe or refractory cases warrants careful exploration, though its safety, efficacy, and mechanisms in pediatric asthma require rigorous evaluation (17, 163). Crucially, these microbiota-targeted strategies must be integrated into the broader context of asthma management as outlined in guidelines like GINA, which emphasize personalized assessment and treatment of modifiable risk factors (11). They should be seen not as replacements but as potential complements or even preventive measures alongside existing pharmacotherapies and biologics (10, 12).

Looking ahead, the field is poised to evolve from a research-centric exploration of associations to a translational discipline capable of informing precision medicine (16, 78). This involves defining asthma endotypes not only by clinical features and inflammatory biomarkers but also by distinct microbial and metabolic signatures (2, 174). By doing so, clinicians may one day be able to predict disease trajectory, tailor interventions, and monitor therapeutic response based on an individual’s unique microbial and immune profile. Achieving this vision will require sustained interdisciplinary collaboration, standardized methodological approaches, and a commitment to translating complex microbiome science into safe, effective, and accessible interventions that can alter the natural history of childhood asthma (17, 185).

## References

[1] Kahhaleh F Barrientos G Conrad M . The gut-lung axis and asthma susceptibility in early life. Acta Physiol. (2024) 240:e14092. doi: 10.1111/apha.14092. PMID: 38251788

[2] Gans M Gavrilova T . Understanding the immunology of asthma: Pathophysiology, biomarkers, and treatments for asthma endotypes. Paediatr Respir Rev. (2020) 36:118–27. doi: 10.1016/j.prrv.2019.08.002. PMID: 31678040

[3] Song P Adeloye D Salim H Dos SJ Campbell H Sheikh A . Global, regional, and national prevalence of asthma in 2019: a systematic analysis and modelling study. J Glob Health. (2022) 12:4052. doi: 10.7189/jogh.12.04052. PMID: 35765786 PMC9239324

[4] Hammad H Lambrecht B . The basic immunology of asthma. Cell. (2021) 184:1469–85. doi: 10.1016/j.cell.2021.02.016. PMID: 33711259

[5] Miyachi H Shibata R Javornik Cregeen SJ Surathu A Sijaric M Espinola JA . Interactions between host genetics and gut microbiome influence susceptibility to childhood asthma and lung function. J Allergy Clin Immunol. (2026) 157:868–78. doi: 10.1016/j.jaci.2025.12.1005. PMID: 41485494 PMC13135221

[6] García-Marcos L Asher M Pearce N Ellwood E Bissell K Chiang C . The burden of asthma, hay fever and eczema in children in 25 countries: GAN Phase I study. Eur Respir J. (2022) 60:2102866. doi: 10.1183/13993003.02866-2021. PMID: 35144987 PMC9474895

[7] Asher M Rutter C Bissell K Chiang C El SA Ellwood E . Worldwide trends in the burden of asthma symptoms in school-aged children: Global Asthma Network Phase I cross-sectional study. Lancet. (2021) 398:1569–80. doi: 10.1016/S0140-6736(21)01450-1. PMID: 34755626 PMC8573635

[8] Zeng X He C Li J Feng Q Lu Z Li L . Gut-lung axis: a novel mechanism involving microbiota dysbiosis-coordinated PLA2-TRPV1 neuroimmune crosstalk in nanoplastic-induced asthma exacerbation. Environ Int. (2026) 207:110047. doi: 10.1016/j.envint.2026.110047. PMID: 41512508

[9] GBD 2019 Diseases and Injuries Collaborators . Global burden of 369 diseases and injuries in 204 countries and territories, 1990-2019: a systematic analysis for the Global Burden of Disease Study 2019. Lancet. (2020) 396:1204–22. doi: 10.1016/S0140-6736(20)30925-9. PMID: 33069326 PMC7567026

[10] Miller R Grayson M Strothman K . Advances in asthma: New understandings of asthma’s natural history, risk factors, underlying mechanisms, and clinical management. J Allergy Clin Immunol. (2021) 148:1430–41. doi: 10.1016/j.jaci.2021.10.001. PMID: 34655640

[11] Reddel H Bacharier L Bateman E Brightling C Brusselle G Buhl R . Global initiative for asthma strategy 2021: executive summary and rationale for key changes. Am J Respir Crit Care Med. (2022) 205:17–35. doi: 10.1164/rccm.202109-2205PP. PMID: 34658302 PMC8865583

[12] Agache I Eguiluz-Gracia I Cojanu C Laculiceanu A Del GS Zemelka-Wiacek M . Advances and highlights in asthma in 2021. Allergy. (2021) 76:3390–407. doi: 10.1111/all.15054. PMID: 34392546

[13] Stern J Pier J Litonjua A . Asthma epidemiology and risk factors. Semin Immunopathol. (2020) 42:5–15. doi: 10.1007/s00281-020-00785-1. PMID: 32020334

[14] Perdijk O Azzoni R Marsland B . The microbiome: an integral player in immune homeostasis and inflammation in the respiratory tract. Physiol Rev. (2024) 104:835–79. doi: 10.1152/physrev.00020.2023. PMID: 38059886

[15] Liu J Hong W Sun Z Zhang S Xue C Dong N . The gut-lung axis: effects and mechanisms of gut microbiota on pulmonary diseases. Front Immunol. (2026) 16:1693964. doi: 10.3389/fimmu.2025.1693964. PMID: 41562083 PMC12812986

[16] Ma Y Zheng H Tian H Cui J Liu L . Research advance in correlation between childhood asthma and gut microbiota. Front Cell Infect Microbiol. (2025) 15:1649180. doi: 10.3389/fcimb.2025.1649180. PMID: 41122685 PMC12536028

[17] Liu L Zhao W Zhang H Shang Y Huang W Cheng Q . Relationship between pediatric asthma and respiratory microbiota, intestinal microbiota: a narrative review. Front Microbiol. (2025) 16:1550783. doi: 10.3389/fmicb.2025.1550783. PMID: 40415934 PMC12099452

[18] Song X Liang J Lin S Xie Y Ke C Ao D . Gut-lung axis and asthma: A historical review on mechanism and future perspective. Clin Transl Allergy. (2024) 14:e12356. doi: 10.1002/clt2.12356. PMID: 38687096 PMC11060082

[19] Zhang D Li S Wang N Tan H Zhang Z Feng Y . The cross-talk between gut microbiota and lungs in common lung diseases. Front Microbiol. (2020) 11:301. doi: 10.3389/fmicb.2020.00301. PMID: 32158441 PMC7052046

[20] Enaud R Prevel R Ciarlo E Beaufils F Wieërs G Guery B . The gut-lung axis in health and respiratory diseases: A place for inter-organ and inter-kingdom crosstalks. Front Cell Infect Microbiol. (2020) 10:9. doi: 10.3389/fcimb.2020.00009. PMID: 32140452 PMC7042389

[21] Barcik W Boutin R Sokolowska M Finlay B . The role of lung and gut microbiota in the pathology of asthma. Immunity. (2020) 52:241–55. doi: 10.1016/j.immuni.2020.01.007. PMID: 32075727 PMC7128389

[22] Wang J Zhu N Su X Gao Y Yang R . Gut-microbiota-derived metabolites maintain gut and systemic immune homeostasis. Cells. (2023) 12:793. doi: 10.3390/cells12050793. PMID: 36899929 PMC10000530

[23] Campbell C Kandalgaonkar M Golonka R Yeoh B Vijay-Kumar M Saha P . Crosstalk between gut microbiota and host immunity: impact on inflammation and immunotherapy. Biomedicines. (2023) 11:294. doi: 10.3390/biomedicines11020294. PMID: 36830830 PMC9953403

[24] Zheng D Liwinski T Elinav E . Interaction between microbiota and immunity in health and disease. Cell Res. (2020) 30:492–506. doi: 10.1038/s41422-020-0332-7. PMID: 32433595 PMC7264227

[25] Yoo J Groer M Dutra S Sarkar A McSkimming D . Gut microbiota and immune system interactions. Microorganisms. (2020) 8:1587. doi: 10.3390/microorganisms8101587. PMID: 33076307 PMC7602490

[26] Paone P Cani P . Mucus barrier, mucins and gut microbiota: the expected slimy partners? Gut. (2020) 69:2232–43. doi: 10.1136/gutjnl-2020-322260. PMID: 32917747 PMC7677487

[27] Huang Y Porsche C Kozik A Lynch S . Microbiome-immune interactions in allergy and asthma. J Allergy Clin Immunol Pr. (2022) 10:2244–51. doi: 10.1016/j.jaip.2022.05.038. PMID: 35724951 PMC10566566

[28] Al NZ Eberl G . Imprinting of the immune system by the microbiota early in life. Mucosal Immunol. (2020) 13:183–9. doi: 10.1038/s41385-020-0257-y. PMID: 31988466

[29] Donald K Finlay B . Early-life interactions between the microbiota and immune system: impact on immune system development and atopic disease. Nat Rev Immunol. (2023) 23:735–48. doi: 10.1038/s41577-023-00874-w. PMID: 37138015

[30] Henrick B Rodriguez L Lakshmikanth T Pou C Henckel E Arzoomand A . Bifidobacteria-mediated immune system imprinting early in life. Cell. (2021) 184:3884–3898.e11. doi: 10.1016/j.cell.2021.05.030. PMID: 34143954

[31] Jacobse J Li J Rings E Samsom J Goettel J . Intestinal regulatory T cells as specialized tissue-restricted immune cells in intestinal immune homeostasis and disease. Front Immunol. (2021) 12:716499. doi: 10.3389/fimmu.2021.716499. PMID: 34421921 PMC8371910

[32] Knoop K McDonald K Hsieh C Tarr P Newberry R . Regulatory T cells developing peri-weaning are continually required to restrain th2 systemic responses later in life. Front Immunol. (2020) 11:603059. doi: 10.3389/fimmu.2020.603059. PMID: 33613522 PMC7891039

[33] Tan J Macia L Mackay C . Dietary fiber and SCFAs in the regulation of mucosal immunity. J Allergy Clin Immunol. (2023) 151:361–70. doi: 10.1016/j.jaci.2022.11.007. PMID: 36543697

[34] Kimura I Ichimura A Ohue-Kitano R Igarashi M . Free fatty acid receptors in health and disease. Physiol Rev. (2020) 100:171–210. doi: 10.1152/physrev.00041.2018. PMID: 31487233

[35] Gasaly N de VP Hermoso M . Impact of bacterial metabolites on gut barrier function and host immunity: A focus on bacterial metabolism and its relevance for intestinal inflammation. Front Immunol. (2021) 12:658354. doi: 10.3389/fimmu.2021.658354. PMID: 34122415 PMC8187770

[36] Yazici D Ogulur I Pat Y Babayev H Barletta E Ardicli S . The epithelial barrier: The gateway to allergic, autoimmune, and metabolic diseases and chronic neuropsychiatric conditions. Semin Immunol. (2023) 70:101846. doi: 10.1016/j.smim.2023.101846. PMID: 37801907

[37] Pattaroni C Marsland B Harris N . Early-life host-microbial interactions and asthma development: A lifelong impact? Immunol Rev. (2025) 330:e70019. doi: 10.1111/imr.70019. PMID: 40099971 PMC11917194

[38] Xiao L Zhao F . Microbial transmission, colonisation and succession: from pregnancy to infancy. Gut. (2023) 72:772–86. doi: 10.1136/gutjnl-2022-328970. PMID: 36720630 PMC10086306

[39] Hoskinson C Dai D Del BK Becker A Moraes T Mandhane P . Delayed gut microbiota maturation in the first year of life is a hallmark of pediatric allergic disease. Nat Commun. (2023) 14:4785. doi: 10.1038/s41467-023-40336-4. PMID: 37644001 PMC10465508

[40] Barnes J Yoshida M He P Worlock K Lindeboom R Suo C . Early human lung immune cell development and its role in epithelial cell fate. Sci Immunol. (2023) 8:eadf9988. doi: 10.1126/sciimmunol.adf9988. PMID: 38100545 PMC7615868

[41] Boulund U Thorsen J Trivedi U Tranæs K Jiang J Shah S . The role of the early-life gut microbiome in childhood asthma. Gut Microbes. (2025) 17:2457489. doi: 10.1080/19490976.2025.2457489. PMID: 39882630 PMC11784655

[42] DeVries A McCauley K Fadrosh D Fujimura K Stern D Lynch S . Maternal prenatal immunity, neonatal trained immunity, and early airway microbiota shape childhood asthma development. Allergy. (2022) 77:3617–28. doi: 10.1111/all.15442. PMID: 35841380 PMC9712226

[43] Gao Y Nanan R Macia L Tan J Sominsky L Quinn T . The maternal gut microbiome during pregnancy and offspring allergy and asthma. J Allergy Clin Immunol. (2021) 148:669–78. doi: 10.1016/j.jaci.2021.07.011. PMID: 34310928

[44] Selma-Royo M García-Mantrana I Calatayud M Parra-Llorca A Martínez-Costa C Collado M . Maternal diet during pregnancy and intestinal markers are associated with early gut microbiota. Eur J Nutr. (2021) 60:1429–42. doi: 10.1007/s00394-020-02337-7. PMID: 32728880

[45] Maher S O’Brien E Moore R Byrne D Geraghty A Saldova R . The association between the maternal diet and the maternal and infant gut microbiome: a systematic review. Br J Nutr. (2023) 129:1491–9. doi: 10.1017/S0007114520000847. PMID: 32129734

[46] Alhasan M Hölsken O Duerr C Helfrich S Branzk N Philipp A . Antibiotic use during pregnancy is linked to offspring gut microbial dysbiosis, barrier disruption, and altered immunity along the gut-lung axis. Eur J Immunol. (2023) 53:e2350394. doi: 10.1002/eji.202350394. PMID: 37431194

[47] Alhasan M Cait A Heimesaat M Blaut M Klopfleisch R Wedel A . Antibiotic use during pregnancy increases offspring asthma severity in a dose-dependent manner. Allergy. (2020) 75:1979–90. doi: 10.1111/all.14234. PMID: 32064643

[48] Gürdeniz G Ernst M Rago D Kim M Courraud J Stokholm J . Neonatal metabolome of caesarean section and risk of childhood asthma. Eur Respir J. (2022) 59:2102406. doi: 10.1183/13993003.02406-2021. PMID: 34887324

[49] Bogaert D van BG de KE Lusarreta PP Balcazar LC Koppensteiner L . Mother-to-infant microbiota transmission and infant microbiota development across multiple body sites. Cell Host Microbe. (2023) 31:447–460.e6. doi: 10.1016/j.chom.2023.01.018. PMID: 36893737

[50] Galazzo G van BN Bervoets L Dapaah I Savelkoul P Hornef M . Development of the microbiota and associations with birth mode, diet, and atopic disorders in a longitudinal analysis of stool samples, collected from infancy through early childhood. Gastroenterology. (2020) 158:1584–96. doi: 10.1053/j.gastro.2020.01.024. PMID: 31958431

[51] Stokholm J Thorsen J Blaser M Rasmussen M Hjelmsø M Shah S . Delivery mode and gut microbial changes correlate with an increased risk of childhood asthma. Sci Transl Med. (2020) 12:eaax9929. doi: 10.1126/scitranslmed.aax9929. PMID: 33177184

[52] Sinha T Prins J Fernández-Pato A Kruk M Dierikx T de MT . Maternal antibiotic prophylaxis during cesarean section has a limited impact on the infant gut microbiome. Cell Host Microbe. (2024) 32:1444–1454.e6. doi: 10.1016/j.chom.2024.07.010. PMID: 39146801 PMC11335186

[53] Depner M Taft D Kirjavainen P Kalanetra K Karvonen A Peschel S . Maturation of the gut microbiome during the first year of life contributes to the protective farm effect on childhood asthma. Nat Med. (2020) 26:1766–75. doi: 10.1038/s41591-020-1095-x. PMID: 33139948

[54] Chen Y Chen Y Lasky-Su J Kelly R Stokholm J Bisgaard H . Environmental and genetic associations with aberrant early-life gut microbial maturation in childhood asthma. J Allergy Clin Immunol. (2023) 151:1494–1502.e14. doi: 10.1016/j.jaci.2023.01.006. PMID: 36649759 PMC10257760

[55] Tsukuda N Yahagi K Hara T Watanabe Y Matsumoto H Mori H . Key bacterial taxa and metabolic pathways affecting gut short-chain fatty acid profiles in early life. ISME J. (2021) 15:2574–90. doi: 10.1038/s41396-021-00937-7. PMID: 33723382 PMC8397723

[56] Patrick D Sbihi H Dai D Al MA Rasali D Rose C . Decreasing antibiotic use, the gut microbiota, and asthma incidence in children: evidence from population-based and prospective cohort studies. Lancet Respir Med. (2020) 8:1094–105. doi: 10.1016/S2213-2600(20)30052-7. PMID: 32220282

[57] Borbet T Pawline M Zhang X Wipperman M Reuter S Maher T . Influence of the early-life gut microbiota on the immune responses to an inhaled allergen. Mucosal Immunol. (2022) 15:1000–11. doi: 10.1038/s41385-022-00544-5. PMID: 35842561 PMC9835105

[58] Zhang Z Wang J Wang H Li Y Jia Y Yi M . Association of infant antibiotic exposure and risk of childhood asthma: a meta-analysis. World Allergy Organ J. (2021) 14:100607. doi: 10.1016/j.waojou.2021.100607. PMID: 34934469 PMC8661061

[59] Christensen E Hjelmsø M Thorsen J Shah S Redgwell T Poulsen C . The developing airway and gut microbiota in early life is influenced by age of older siblings. Microbiome. (2022) 10:106. doi: 10.1186/s40168-022-01305-z. PMID: 35831879 PMC9277889

[60] Luukkonen J Moustgaard H Martikainen P Remes H . Does having siblings really protect against childhood atopic diseases? A total population and within-family analysis. Eur J Epidemiol. (2024) 39:289–98. doi: 10.1007/s10654-024-01104-w. PMID: 38316709 PMC10995035

[61] Ning Z Zhang Y Lu R Zhao A Wang Z Yuan J . The association between prenatal exposure and childhood asthma: the mediating role of gut microbiota. Front Microbiol. (2025) 16:1664708. doi: 10.3389/fmicb.2025.1664708. PMID: 41040870 PMC12484141

[62] Panzer A Sitarik A Fadrosh D Havstad S Jones K Davidson B . The impact of prenatal dog keeping on infant gut microbiota development. Clin Exp Allergy. (2023) 53:833–45. doi: 10.1111/cea.14303. PMID: 36916778 PMC11163251

[63] Pattaroni C Macowan M Chatzis R Daunt C Custovic A Shields M . Early life inter-kingdom interactions shape the immunological environment of the airways. Microbiome. (2022) 10:34. doi: 10.1186/s40168-021-01201-y. PMID: 35189979 PMC8862481

[64] Cukrowska B Bierła J Zakrzewska M Klukowski M Maciorkowska E . The relationship between the infant gut microbiota and allergy. The role of Bifidobacterium breve and prebiotic oligosaccharides in the activation of anti-allergic mechanisms in early life. Nutrients. (2020) 12:946. doi: 10.3390/nu12040946. PMID: 32235348 PMC7230322

[65] Sindi A Geddes D Wlodek M Muhlhausler B Payne M Stinson L . Can we modulate the breastfed infant gut microbiota through maternal diet? FEMS Microbiol Rev. (2021) 45:fuab011. doi: 10.1093/femsre/fuab011. PMID: 33571360

[66] Leal RC Shah S Rasmussen M Thorsen J Boulund U Pedersen C . The infant gut virome is associated with preschool asthma risk independently of bacteria. Nat Med. (2024) 30:138–48. doi: 10.1038/s41591-023-02685-x. PMID: 38102298

[67] Fang Z Stickley S Ambalavanan A Zhang Y Zacharias A Fehr K . Networks of human milk microbiota are associated with host genomics, childhood asthma, and allergic sensitization. Cell Host Microbe. (2024) 32:1838–1852.e5. doi: 10.1016/j.chom.2024.08.014. PMID: 39293435

[68] Kotrba J Müller I Pausder A Hoffmann A Camp B Boehme J . Innate players in Th2 and non-Th2 asthma: emerging roles for the epithelial cell, mast cell, and monocyte/macrophage network. Am J Physiol Cell Physiol. (2024) 327:C1373–83. doi: 10.1152/ajpcell.00488.2024. PMID: 39401422

[69] Di GA Di CM Comberiati P Peroni D . Go with your gut: the shaping of T-cell response by gut microbiota in allergic asthma. Front Immunol. (2020) 11:1485. doi: 10.3389/fimmu.2020.01485. PMID: 32760404 PMC7372123

[70] Matia-Garcia I Vadillo E Pelayo R Muñoz-Valle J García-Chagollán M Loaeza-Loaeza J . Th1/th2 balance in young subjects: relationship with cytokine levels and metabolic profile. J Inflammation Res. (2021) 14:6587–600. doi: 10.2147/JIR.S342545. PMID: 34908860 PMC8664383

[71] Losol P Sokolowska M Hwang Y Ogulur I Mitamura Y Yazici D . Epithelial barrier theory: the role of exposome, microbiome, and barrier function in allergic diseases. Allergy Asthma Immunol Res. (2023) 15:705–24. doi: 10.4168/aair.2023.15.6.705. PMID: 37957791 PMC10643858

[72] Akdis C . Does the epithelial barrier hypothesis explain the increase in allergy, autoimmunity and other chronic conditions? Nat Rev Immunol. (2021) 21:739–51. doi: 10.1038/s41577-021-00538-7. PMID: 33846604

[73] Thorsen J Li X Peng S Sunde R Shah S Bhattacharyya M . The airway microbiota of neonates colonized with asthma-associated pathogenic bacteria. Nat Commun. (2023) 14:6668. doi: 10.1038/s41467-023-42309-z. PMID: 37863895 PMC10589220

[74] Sunde R Thorsen J Kim M Schoos A Stokholm J Bønnelykke K . Bacterial colonisation of the airway in neonates and risk of asthma and allergy until age 18 years. Eur Respir J. (2024) 63:2300471. doi: 10.1183/13993003.00471-2023. PMID: 38097209

[75] Tang H Lang A Teo S Judd L Gangnon R Evans M . Developmental patterns in the nasopharyngeal microbiome during infancy are associated with asthma risk. J Allergy Clin Immunol. (2021) 147:1683–91. doi: 10.1016/j.jaci.2020.10.009. PMID: 33091409 PMC7571460

[76] de SPW Watson R de KE Hasrat R Arp K Chu M . Early-life viral infections are associated with disadvantageous immune and microbiota profiles and recurrent respiratory infections. Nat Microbiol. (2022) 7:224–37. doi: 10.1038/s41564-021-01043-2. PMID: 35058634

[77] Melgaard M Jensen S Eliasen A Pedersen C Thorsen J Mikkelsen M . Asthma development is associated with low mucosal IL-10 during viral infections in early life. Allergy. (2024) 79:2981–92. doi: 10.1111/all.16276. PMID: 39221476

[78] Liu C Makrinioti H Saglani S Bowman M Lin L Camargo CJ . Microbial dysbiosis and childhood asthma development: integrated role of the airway and gut microbiome, environmental exposures, and host metabolic and immune response. Front Immunol. (2022) 13:1028209. doi: 10.3389/fimmu.2022.1028209. PMID: 36248891 PMC9561420

[79] Hufnagl K Pali-Schöll I Roth-Walter F Jensen-Jarolim E . Dysbiosis of the gut and lung microbiome has a role in asthma. Semin Immunopathol. (2020) 42:75–93. doi: 10.1007/s00281-019-00775-y. PMID: 32072252 PMC7066092

[80] Kim Y Park M Kim S Kim M Kim K Sohn M . Respiratory microbiome profiles are associated with distinct inflammatory phenotype and lung function in children with asthma. J Investig Allergol Clin Immunol. (2024) 34:246–56. doi: 10.18176/jiaci.0918. PMID: 37260034

[81] McCauley K Flynn K Calatroni A DiMassa V LaMere B Fadrosh D . Seasonal airway microbiome and transcriptome interactions promote childhood asthma exacerbations. J Allergy Clin Immunol. (2022) 150:204–13. doi: 10.1016/j.jaci.2022.01.020. PMID: 35149044

[82] Yuan H Liu Z Dong J Bacharier L Jackson D Mauger D . The fungal microbiome of the upper airway is associated with future loss of asthma control and exacerbation among children with asthma. Chest. (2023) 164:302–13. doi: 10.1016/j.chest.2023.03.034. PMID: 37003356 PMC10477953

[83] Choi S Sohn K Jung J Kang M Yang M Kim S . Lung virome: new potential biomarkers for asthma severity and exacerbation. J Allergy Clin Immunol. (2021) 148:1007–1015.e9. doi: 10.1016/j.jaci.2021.03.017. PMID: 33757721

[84] Megremis S Constantinides B Xepapadaki P Yap C Sotiropoulos A Bachert C . Respiratory eukaryotic virome expansion and bacteriophage deficiency characterize childhood asthma. Sci Rep. (2023) 13:8319. doi: 10.1038/s41598-023-34730-7. PMID: 37221274 PMC10205716

[85] Zheng P Zhang K Lv X Liu C Wang Q Bai X . Gut microbiome and metabolomics profiles of allergic and non-allergic childhood asthma. J Asthma Allergy. (2022) 15:419–35. doi: 10.2147/JAA.S354870. PMID: 35418758 PMC8995180

[86] Lee-Sarwar K Dedrick S Momeni B Kelly R Zeiger R O’Connor G . Association of the gut microbiome and metabolome with wheeze frequency in childhood asthma. J Allergy Clin Immunol. (2022) 150:325–36. doi: 10.1016/j.jaci.2022.02.005. PMID: 35196534 PMC9359927

[87] Wilson N Hernandez-Leyva A Rosen A Jaeger N McDonough R Santiago-Borges J . The gut microbiota of people with asthma influences lung inflammation in gnotobiotic mice. iScience. (2023) 26:105991. doi: 10.1016/j.isci.2023.105991. PMID: 36824270 PMC9941210

[88] Mahdavinia M Fyolek J Jiang J Thivalapill N Bilaver L Warren C . Gut microbiome is associated with asthma and race in children with food allergy. J Allergy Clin Immunol. (2023) 152:1541–1549.e1. doi: 10.1016/j.jaci.2023.07.024. PMID: 37714436 PMC10872992

[89] Mansbach J Luna P Shaw C Hasegawa K Petrosino J Piedra P . Increased Moraxella and Streptococcus species abundance after severe bronchiolitis is associated with recurrent wheezing. J Allergy Clin Immunol. (2020) 145:518–527.e8. doi: 10.1016/j.jaci.2019.10.034. PMID: 31738994 PMC7010548

[90] Rosas-Salazar C Chirkova T Gebretsadik T Chappell J Peebles RJ Dupont W . Respiratory syncytial virus infection during infancy and asthma during childhood in the USA (INSPIRE): a population-based, prospective birth cohort study. Lancet. (2023) 401:1669–80. doi: 10.1016/S0140-6736(23)00811-5. PMID: 37086744 PMC10367596

[91] Shi T Li N He Y Feng J Mei Z Du Y . Th17/Treg cell imbalance plays an important role in respiratory syncytial virus infection compromising asthma tolerance in mice. Microb Pathog. (2021) 156:104867. doi: 10.1016/j.micpath.2021.104867. PMID: 33957244

[92] Wu B Sulaiman I Tsay J Perez L Franca B Li Y . Episodic aspiration with oral commensals induces a MyD88-dependent, pulmonary T-helper cell type 17 response that mitigates susceptibility to Streptococcus pneumoniae. Am J Respir Crit Care Med. (2021) 203:1099–111. doi: 10.1164/rccm.202005-1596OC. PMID: 33166473 PMC8314894

[93] Lee-Sarwar K Chen Y Chen Y Kozyrskyj A Mandhane P Turvey S . The maternal prenatal and offspring early-life gut microbiome of childhood asthma phenotypes. Allergy. (2023) 78:418–28. doi: 10.1111/all.15516. PMID: 36107703 PMC9892205

[94] James B Oyeniran C Sturgill J Newton J Martin R Bieberich E . Ceramide in apoptosis and oxidative stress in allergic inflammation and asthma. J Allergy Clin Immunol. (2021) 147:1936–1948.e9. doi: 10.1016/j.jaci.2020.10.024. PMID: 33130063 PMC8081742

[95] Shin S Cho K Yoon H Kim S Kim H Pewzner-Jung Y . Ceramide synthase 2 null mice are protected from ovalbumin-induced asthma with higher T cell receptor signal strength in CD4+ T cells. Int J Mol Sci. (2021) 22:2713. doi: 10.3390/ijms22052713. PMID: 33800208 PMC7962461

[96] Vandenborght L Enaud R Urien C Coron N Girodet P Ferreira S . Type 2-high asthma is associated with a specific indoor mycobiome and microbiome. J Allergy Clin Immunol. (2021) 147:1296–1305.e6. doi: 10.1016/j.jaci.2020.08.035. PMID: 32926879 PMC7486598

[97] Lagree K Underhill D . Candida-induced asthma steps up to the plate-lets. Immunity. (2021) 54:2442–4. doi: 10.1016/j.immuni.2021.10.014. PMID: 34758334

[98] Curren B Ahmed T Rashid R Sebina I Al ASM Howard D . A maternal high-fat diet predisposes to infant lung disease via increased neutrophil-mediated IL-6 trans-signaling. Cell Rep. (2024) 43:114974. doi: 10.1016/j.celrep.2024.114974. PMID: 39535919

[99] Kong J Yang F Zong Y Wang M Jiang S Ma Z . Early-life antibiotic exposure promotes house dust mite-induced allergic airway inflammation by impacting gut microbiota and lung lipid metabolism. Int Immunopharmacol. (2024) 128:111449. doi: 10.1016/j.intimp.2023.111449. PMID: 38199196

[100] Ubags N Trompette A Pernot J Nibbering B Wong N Pattaroni C . Microbiome-induced antigen-presenting cell recruitment coordinates skin and lung allergic inflammation. J Allergy Clin Immunol. (2021) 147:1049–1062.e7. doi: 10.1016/j.jaci.2020.06.030. PMID: 32679208

[101] Raita Y Camargo CJ Bochkov Y Celedón J Gern J Mansbach J . Integrated-omics endotyping of infants with rhinovirus bronchiolitis and risk of childhood asthma. J Allergy Clin Immunol. (2021) 147:2108–17. doi: 10.1016/j.jaci.2020.11.002. PMID: 33197460 PMC8116357

[102] Durack J Christian L Nariya S Gonzalez J Bhakta N Ansel K . Distinct associations of sputum and oral microbiota with atopic, immunologic, and clinical features in mild asthma. J Allergy Clin Immunol. (2020) 146:1016–26. doi: 10.1016/j.jaci.2020.03.028. PMID: 32298699 PMC7554083

[103] Diver S Haldar K McDowell P Busby J Mistry V Micieli C . Relationship between inflammatory status and microbial composition in severe asthma and during exacerbation. Allergy. (2022) 77:3362–76. doi: 10.1111/all.15425. PMID: 35778780

[104] van der Hb Wells J . Microbial regulation of host physiology by short-chain fatty acids. Trends Microbiol. (2021) 29:700–12. doi: 10.1016/j.tim.2021.02.001. PMID: 33674141

[105] Deleu S Machiels K Raes J Verbeke K Vermeire S . Short chain fatty acids and its producing organisms: An overlooked therapy for IBD? EBioMedicine. (2021) 66:103293. doi: 10.1016/j.ebiom.2021.103293. PMID: 33813134 PMC8047503

[106] Yip W Hughes M Li Y Cait A Hirst M Mohn W . Butyrate shapes immune cell fate and function in allergic asthma. Front Immunol. (2021) 12:628453. doi: 10.3389/fimmu.2021.628453. PMID: 33659009 PMC7917140

[107] Huang C Du W Ni Y Lan G Shi G . The effect of short-chain fatty acids on M2 macrophages polarization *in vitro* and *in vivo*. Clin Exp Immunol. (2022) 207:53–64. doi: 10.1093/cei/uxab028. PMID: 35020860 PMC8802183

[108] Trompette A Pernot J Perdijk O Alqahtani R Domingo J Camacho-Muñoz D . Gut-derived short-chain fatty acids modulate skin barrier integrity by promoting keratinocyte metabolism and differentiation. Mucosal Immunol. (2022) 15:908–26. doi: 10.1038/s41385-022-00524-9. PMID: 35672452 PMC9385498

[109] Campbell C McKenney P Konstantinovsky D Isaeva O Schizas M Verter J . Bacterial metabolism of bile acids promotes generation of peripheral regulatory T cells. Nature. (2020) 581:475–9. doi: 10.1038/s41586-020-2193-0. PMID: 32461639 PMC7540721

[110] Hu M Alashkar AB Santner-Nanan B Miethe S Harb H Renz H . Short-chain fatty acids augment differentiation and function of human induced regulatory T cells. Int J Mol Sci. (2022) 23:5740. doi: 10.3390/ijms23105740. PMID: 35628549 PMC9143307

[111] Kim H Kim B Holzapfel W Kang H . Lactiplantibacillusplantarum APsulloc331261 (GTB1(^TM^)) promotes butyrate production to suppress mucin hypersecretion in a murine allergic airway inflammation model. Front Microbiol. (2023) 14:1292266. doi: 10.3389/fmicb.2023.1292266. PMID: 38449878 PMC10915089

[112] Bezemer G Diks M Mortaz E van Ai van Bj Kraneveld A . A synbiotic mixture of Bifidobacterium breve M16-V, oligosaccharides and pectin, enhances short chain fatty acid production and improves lung health in a preclinical model for pulmonary neutrophilia. Front Nutr. (2024) 11:1371064. doi: 10.3389/fnut.2024.1371064. PMID: 39006103 PMC11239554

[113] Lai Y Qiu R Zhou J Ren L Qu Y Zhang G . Fecal microbiota transplantation alleviates airway inflammation in asthmatic rats by increasing the level of short-chain fatty acids in the intestine. Inflammation. (2025) 48:1538–52. doi: 10.1007/s10753-024-02233-w. PMID: 39775370 PMC12234594

[114] Laursen M Sakanaka M von Bn Mörbe U Andersen D Moll J . Bifidobacterium species associated with breastfeeding produce aromatic lactic acids in the infant gut. Nat Microbiol. (2021) 6:1367–82. doi: 10.1038/s41564-021-00970-4. PMID: 34675385 PMC8556157

[115] Yu K Li Q Sun X Peng X Tang Q Chu H . Bacterial indole-3-lactic acid affects epithelium-macrophage crosstalk to regulate intestinal homeostasis. Proc Natl Acad Sci U A. (2023) 120:e2309032120. doi: 10.1073/pnas.2309032120. PMID: 37903267 PMC10636326

[116] Ehrlich A Pacheco A Henrick B Taft D Xu G Huda M . Indole-3-lactic acid associated with Bifidobacterium-dominated microbiota significantly decreases inflammation in intestinal epithelial cells. BMC Microbiol. (2020) 20:357. doi: 10.1186/s12866-020-02023-y. PMID: 33225894 PMC7681996

[117] Wang H He Y Dang D Feng L Huang L Zhao J . Bifidobacterium animalis subsp. lactis CCFM1274 relieved allergic asthma symptoms by modifying intestinal tryptophan metabolism in mice. Food Funct. (2024) 15:8810–22. doi: 10.1039/d4fo01079e. PMID: 39115430

[118] Perdijk O Butler A Macowan M Chatzis R Bulanda E Grant R . Antibiotic-driven dysbiosis in early life disrupts indole-3-propionic acid production and exacerbates allergic airway inflammation in adulthood. Immunity. (2024) 57:1939–1954.e7. doi: 10.1016/j.immuni.2024.06.010. PMID: 39013465

[119] Hu Q Jin L Zeng J Wang J Zhong S Fan W . Tryptophan metabolite-regulated Treg responses contribute to attenuation of airway inflammation during specific immunotherapy in a mouse asthma model. Hum Vaccin Immunother. (2020) 16:1891–9. doi: 10.1080/21645515.2019.1698900. PMID: 31951781 PMC7482871

[120] Sanidad K Rager S Carrow H Ananthanarayanan A Callaghan R Hart L . Gut bacteria-derived serotonin promotes immune tolerance in early life. Sci Immunol. (2024) 9:eadj4775. doi: 10.1126/sciimmunol.adj4775. PMID: 38489352 PMC11328322

[121] Arifuzzaman M Won T Li T Yano H Digumarthi S Heras A . Inulin fibre promotes microbiota-derived bile acids and type 2 inflammation. Nature. (2022) 611:578–84. doi: 10.1038/s41586-022-05380-y. PMID: 36323778 PMC10576985

[122] Maguire T Yung S Ortiz-Zapater E Kayode O Till S Corrigan C . Sphingosine-1-phosphate induces airway smooth muscle hyperresponsiveness and proliferation. J Allergy Clin Immunol. (2023) 152:1131–1140.e6. doi: 10.1016/j.jaci.2023.05.028. PMID: 37474025

[123] Pujo J Petitfils C Le Fp Eeckhaut V Payros G Maurel S . Bacteria-derived long chain fatty acid exhibits anti-inflammatory properties in colitis. Gut. (2021) 70:1088–97. doi: 10.1136/gutjnl-2020-321173. PMID: 32978245

[124] Romaní-Pérez M López-Almela I Bullich-Vilarrubias C Rueda-Ruzafa L Gómez Dpe Benítez-Páez A . Holdemanella biformis improves glucose tolerance and regulates GLP-1 signaling in obese mice. FASEB J. (2021) 35:e21734. doi: 10.1096/fj.202100126R. PMID: 34143451

[125] Lee J Hall J Kroehling L Wu L Najar T Nguyen H . Serum amyloid A proteins induce pathogenic Th17 cells and promote inflammatory disease. Cell. (2020) 180:79–91.e16. doi: 10.1016/j.cell.2019.11.026. PMID: 31866067 PMC7039443

[126] Sanchez H Moroney J Gan H Shen T Im J Li T . B cell-intrinsic epigenetic modulation of antibody responses by dietary fiber-derived short-chain fatty acids. Nat Commun. (2020) 11:60. doi: 10.1038/s41467-019-13603-6. PMID: 31896754 PMC6940392

[127] Hsieh C Rengarajan S Kau A Tarazona-Meza C Nicholson A Checkley W . Altered IgA response to gut bacteria is associated with childhood asthma in Peru. J Immunol. (2021) 207:398–407. doi: 10.4049/jimmunol.2001296. PMID: 34193598 PMC8516662

[128] Rowland S Green C Halliwill J Singanayagam A Heaney L . Gut feelings on short-chain fatty acids to regulate respiratory health. Trends Endocrinol Metab. (2025) 36:889–98. doi: 10.1016/j.tem.2024.12.007. PMID: 39757060

[129] Alharris E Mohammed A Alghetaa H Zhou J Nagarkatti M Nagarkatti P . The ability of resveratrol to attenuate ovalbumin-mediated allergic asthma is associated with changes in microbiota involving the gut-lung axis, enhanced barrier function and decreased inflammation in the lungs. Front Immunol. (2022) 13:805770. doi: 10.3389/fimmu.2022.805770. PMID: 35265071 PMC8898895

[130] Brustad N Yang L Chawes B Stokholm J Gürdeniz G Bønnelykke K . Fish oil and vitamin D supplementations in pregnancy protect against childhood croup. J Allergy Clin Immunol Pr. (2023) 11:315–21. doi: 10.1016/j.jaip.2022.09.027. PMID: 36184023

[131] Lee E Park Y Lee S Lee S Park M Ahn K . Associations of prenatal antibiotic exposure and delivery mode on childhood asthma inception. Ann Allergy Asthma Immunol. (2023) 131:52–58.e1. doi: 10.1016/j.anai.2023.03.020. PMID: 36990205

[132] Pechlivanis S Depner M Kirjavainen P Roduit C Täubel M Frei R . Continuous rather than solely early farm exposure protects from hay fever development. J Allergy Clin Immunol Pr. (2023) 11:591–601. doi: 10.1016/j.jaip.2022.10.035. PMID: 36356926 PMC9907754

[133] Brick T Hettinga K Kirchner B Pfaffl M Ege M . The beneficial effect of farm milk consumption on asthma, allergies, and infections: From meta-analysis of evidence to clinical trial. J Allergy Clin Immunol Pr. (2020) 8:878–889.e3. doi: 10.1016/j.jaip.2019.11.017. PMID: 31770653

[134] Lehtimäki J Thorsen J Rasmussen M Hjelmsø M Shah S Mortensen M . Urbanized microbiota in infants, immune constitution, and later risk of atopic diseases. J Allergy Clin Immunol. (2021) 148:234–43. doi: 10.1016/j.jaci.2020.12.621. PMID: 33338536

[135] Zhang M Tang H Chen Y Chen Z Xu Y Fu X . Impact of environmental characteristics on children’s gut microbiota - A pilot study in assessing the role of indoor microbiome and metabolites. Env Res. (2023) 234:116114. doi: 10.1016/j.envres.2023.116114. PMID: 37209986

[136] Kurilshikov A Medina-Gomez C Bacigalupe R Radjabzadeh D Wang J Demirkan A . Large-scale association analyses identify host factors influencing human gut microbiome composition. Nat Genet. (2021) 53:156–65. doi: 10.1038/s41588-020-00763-1. PMID: 33462485 PMC8515199

[137] Vandenplas Y Żołnowska M Berni Cr Ludman S Tengelyi Z Moreno-Álvarez A . Effects of an extensively hydrolyzed formula supplemented with two human milk oligosaccharides on growth, tolerability, safety and infection risk in infants with cow’s milk protein allergy: A randomized, multi-center trial. Nutrients. (2022) 14:530. doi: 10.3390/nu14030530. PMID: 35276889 PMC8839689

[138] Saeed M Gribben K Alam M Lyden E Hanson C Levan T . Association of dietary fiber on asthma, respiratory symptoms, and inflammation in the adult National Health and Nutrition Examination Survey population. Ann Am Thorac Soc. (2020) 17:1062–8. doi: 10.1513/AnnalsATS.201910-776OC. PMID: 32369709

[139] Venter C Meyer R Greenhawt M Pali-Schöll I Nwaru B Roduit C . Role of dietary fiber in promoting immune health-an EAACI position paper. Allergy. (2022) 77:3185–98. doi: 10.1111/all.15430. PMID: 35801383

[140] Vázquez-Cuesta S Lozano Gn Rodríguez-Fernández S Fernández-Avila A Bermejo J Fernández-Avilés F . Impact of the Mediterranean diet on the gut microbiome of a well-defined cohort of healthy individuals. Nutrients. (2024) 16:793. doi: 10.3390/nu16060793. PMID: 38542704 PMC10974552

[141] Li X Brejnrod A Thorsen J Zachariasen T Trivedi U Russel J . Differential responses of the gut microbiome and resistome to antibiotic exposures in infants and adults. Nat Commun. (2023) 14:8526. doi: 10.1038/s41467-023-44289-6. PMID: 38135681 PMC10746713

[142] Boutin R Sbihi H McLaughlin R Hahn A Konwar K Loo R . Composition and associations of the infant gut fungal microbiota with environmental factors and childhood allergic outcomes. mBio. (2021) 12:e0339620. doi: 10.1128/mBio.03396-20. PMID: 34060330 PMC8263004

[143] Hu L Ni Z Zhao K Li X Gao X Kang Y . The association between oral and gut microbiota in male patients with alcohol dependence. Front Microbiol. (2023) 14:1203678. doi: 10.3389/fmicb.2023.1203678. PMID: 37577447 PMC10422022

[144] Ye C Katagiri S Miyasaka N Kobayashi H Khemwong T Nagasawa T . The periodontopathic bacteria in placenta, saliva and subgingival plaque of threatened preterm labor and preterm low birth weight cases: a longitudinal study in Japanese pregnant women. Clin Oral Investig. (2020) 24:4261–70. doi: 10.1007/s00784-020-03287-4. PMID: 32333174

[145] Rubio-Portillo E Orts D Llorca E Fernández C Antón J Ferrer C . The domestic environment and the lung mycobiome. Microorganisms. (2020) 8:1717. doi: 10.3390/microorganisms8111717. PMID: 33147738 PMC7693370

[146] Cheng Z Wu Y Jie Z Li X Zhang J . Genetic evidence on the causality between gut microbiota and various asthma phenotypes: a two-sample Mendelian randomization study. Front Cell Infect Microbiol. (2023) 13:1270067. doi: 10.3389/fcimb.2023.1270067. PMID: 38274730 PMC10808785

[147] Shi H Zhao T Geng R Sun L Fan H . The associations between gut microbiota and chronic respiratory diseases: a Mendelian randomization study. Front Microbiol. (2023) 14:1200937. doi: 10.3389/fmicb.2023.1200937. PMID: 37333634 PMC10272395

[148] Li R Guo Q Zhao J Kang W Lu R Long Z . Assessing causal relationships between gut microbiota and asthma: evidence from two sample Mendelian randomization analysis. Front Immunol. (2023) 14:1148684. doi: 10.3389/fimmu.2023.1148684. PMID: 37539057 PMC10394653

[149] Gagnon E Mitchell P Manikpurage H Abner E Taba N Esko T . Impact of the gut microbiota and associated metabolites on cardiometabolic traits, chronic diseases and human longevity: a Mendelian randomization study. J Transl Med. (2023) 21:60. doi: 10.1186/s12967-022-03799-5. PMID: 36717893 PMC9887809

[150] Fan D Hu J Lin N . Effects of probiotics, prebiotics, synbiotics and postbiotics on pediatric asthma: a systematic review. Front Nutr. (2025) 12:1586129. doi: 10.3389/fnut.2025.1586129. PMID: 40352259 PMC12061971

[151] Drago L Cioffi L Giuliano M Pane M Amoruso A Schiavetti I . The Probiotics in Pediatric Asthma Management (PROPAM) Study in the Primary Care Setting: A Randomized, Controlled, Double-Blind Trial with Ligilactobacillus salivarius LS01 (DSM 22775) and Bifidobacterium breve B632 (DSM 24706). J Immunol Res. (2022) 2022:3837418. doi: 10.1155/2022/3837418. PMID: 35083341 PMC8786459

[152] Liu A Ma T Xu N Jin H Zhao F Kwok L . Adjunctive probiotics alleviates asthmatic symptoms via modulating the gut microbiome and serum metabolome. Microbiol Spectr. (2021) 9:e0085921. doi: 10.1128/Spectrum.00859-21. PMID: 34612663 PMC8510161

[153] Chen X Yong S Yii C Feng B Hsieh K Li Q . Intestinal microbiota and probiotic intervention in children with bronchial asthma. Heliyon. (2024) 10:e34916. doi: 10.1016/j.heliyon.2024.e34916. PMID: 39144926 PMC11320201

[154] Li L Fang Z Lee Y Zhao J Zhang H Lu W . Prophylactic effects of oral administration of Lactobacillus casei on house dust mite-induced asthma in mice. Food Funct. (2020) 11:9272–84. doi: 10.1039/d0fo01363c. PMID: 33047743

[155] Pyclik M Srutkova D Razim A Hermanova P Svabova T Pacyga K . Viability status-dependent effect of Bifidobacterium longum ssp. longum CCM 7952 on prevention of allergic inflammation in mouse model. Front Immunol. (2021) 12:707728. doi: 10.3389/fimmu.2021.707728. PMID: 34354710 PMC8329652

[156] Idrees M Imran M Atiq N Zahra R Abid R Alreshidi M . Probiotics, their action modality and the use of multi-omics in metamorphosis of commensal microbiota into target-based probiotics. Front Nutr. (2022) 9:959941. doi: 10.3389/fnut.2022.959941. PMID: 36185680 PMC9523698

[157] Salminen S Collado M Endo A Hill C Lebeer S Quigley E . The International Scientific Association of Probiotics and Prebiotics (ISAPP) consensus statement on the definition and scope of postbiotics. Nat Rev Gastroenterol Hepatol. (2021) 18:649–67. doi: 10.1038/s41575-021-00440-6. PMID: 33948025 PMC8387231

[158] Mi X Tran T Park H Xu X Subramaniyam S Choi H . Immune-enhancing effects of postbiotic produced by Bacillus velezensis Kh2–2 isolated from Korea Foods. Food Res Int. (2022) 152:110911. doi: 10.1016/j.foodres.2021.110911. PMID: 35181083

[159] Li L Sun Q Xiao H Zhang Q Xu S Lai L . Aerosol inhalation of heat-killed Clostridium butyricum CGMCC0313–1 alleviates allergic airway inflammation in mice. J Immunol Res. (2022) 2022:8447603. doi: 10.1155/2022/8447603. PMID: 36033385 PMC9410851

[160] Węgrzyn K Jasińska A Janeczek K Feleszko W . The role of postbiotics in asthma treatment. Microorganisms. (2024) 12:1642. doi: 10.3390/microorganisms12081642. PMID: 39203484 PMC11356534

[161] Palmer D Cuthbert A Sullivan T Pretorius R Garssen J Rueter K . Effects of pregnancy and lactation prebiotics supplementation on infant allergic disease: a randomized controlled trial. J Allergy Clin Immunol. (2025) 155:144–52. doi: 10.1016/j.jaci.2024.08.009. PMID: 39173718

[162] Zheng J Huang Y Zhang L Liu T Zou Y He L . Role of the gut-lung microbiome axis in airway inflammation in OVA-challenged mice and the effect of azithromycin. J Inflammation Res. (2025) 18:2661–76. doi: 10.2147/JIR.S506688. PMID: 40008084 PMC11853874

[163] Yadegar A Bar-Yoseph H Monaghan T Pakpour S Severino A Kuijper E . Fecal microbiota transplantation: current challenges and future landscapes. Clin Microbiol Rev. (2024) 37:e0006022. doi: 10.1128/cmr.00060-22. PMID: 38717124 PMC11325845

[164] Mogoş G Manciulea PM Enache R Pavelescu L Popescu RO Cretoiu S . Intestinal microbiota in early life: latest findings regarding the role of probiotics as a treatment approach for dysbiosis. Nutrients. (2025) 17:2071. doi: 10.3390/nu17132071. PMID: 40647176 PMC12250897

[165] Colquitt A Miles E Calder P . Do probiotics in pregnancy reduce allergies and asthma in infancy and childhood? A systematic review. Nutrients. (2022) 14:1852. doi: 10.3390/nu14091852. PMID: 35565819 PMC9105059

[166] Meirlaen L Levy E Vandenplas Y . Prevention and management with pro-, pre and synbiotics in children with asthma and allergic rhinitis: a narrative review. Nutrients. (2021) 13:934. doi: 10.3390/nu13030934. PMID: 33799367 PMC7999316

[167] Lejeune S Deschildre A Le RO Engelmann I Dessein R Pichavant M . Childhood asthma heterogeneity at the era of precision medicine: modulating the immune response or the microbiota for the management of asthma attack. Biochem Pharmacol. (2020) 179:114046. doi: 10.1016/j.bcp.2020.114046. PMID: 32446884 PMC7242211

[168] Hudey S Ledford D Cardet J . Mechanisms of non-type 2 asthma. Curr Opin Immunol. (2020) 66:123–8. doi: 10.1016/j.coi.2020.10.002. PMID: 33160187 PMC7852882

[169] Akar-Ghibril N Casale T Custovic A Phipatanakul W . Allergic endotypes and phenotypes of asthma. J Allergy Clin Immunol Pr. (2020) 8:429–40. doi: 10.1016/j.jaip.2019.11.008. PMID: 32037107 PMC7569362

[170] Hachim M Alqutami F Hachim I Heialy S Busch H Hamoudi R . The role of systems biology in deciphering asthma heterogeneity. Life Basel. (2022) 12:1562. doi: 10.3390/life12101562. PMID: 36294997 PMC9605413

[171] Kloepfer K Kennedy J . Childhood respiratory viral infections and the microbiome. J Allergy Clin Immunol. (2023) 152:827–34. doi: 10.1016/j.jaci.2023.08.008. PMID: 37607643 PMC10592030

[172] Chun Y Do A Grishina G Grishin A Fang G Rose S . Integrative study of the upper and lower airway microbiome and transcriptome in asthma. JCI Insight. (2020) 5:e133707. doi: 10.1172/jci.insight.133707. PMID: 32161195 PMC7141394

[173] Chiu C Chang K Chang L Wang C Chung W Hsieh W . Phenotype-specific signatures of systems-level gut microbiome associated with childhood airway allergies. Pediatr Allergy Immunol. (2023) 34:e13905. doi: 10.1111/pai.13905. PMID: 36705037

[174] Macowan M Pattaroni C Bonner K Chatzis R Daunt C Gore M . Deep multiomic profiling reveals molecular signatures that underpin preschool wheeze and asthma. J Allergy Clin Immunol. (2025) 155:94–106. doi: 10.1016/j.jaci.2024.08.017. PMID: 39214237

[175] Cobos-Uribe C Rebuli M . Understanding the functional role of the microbiome and metabolome in asthma. Curr Allergy Asthma Rep. (2023) 23:67–76. doi: 10.1007/s11882-022-01056-9. PMID: 36525159 PMC12340730

[176] Spakowicz D Lou S Barron B Gomez J Li T Liu Q . Approaches for integrating heterogeneous RNA-seq data reveal cross-talk between microbes and genes in asthmatic patients. Genome Biol. (2020) 21:150. doi: 10.1186/s13059-020-02033-z. PMID: 32571363 PMC7310008

[177] Huang C Yu Y Du W Liu Y Dai R Tang W . Fungal and bacterial microbiome dysbiosis and imbalance of trans-kingdom network in asthma. Clin Transl Allergy. (2020) 10:42. doi: 10.1186/s13601-020-00345-8. PMID: 33110490 PMC7583303

[178] Wu G Xu T Zhao N Lam Y Ding X Wei D . A core microbiome signature as an indicator of health. Cell. (2024) 187:6550–6565.e11. doi: 10.1016/j.cell.2024.09.019. PMID: 39378879

[179] Lee-Sarwar K Lasky-Su J Kelly R Litonjua A Weiss S . Gut microbial-derived metabolomics of asthma. Metabolites. (2020) 10:97. doi: 10.3390/metabo10030097. PMID: 32155960 PMC7142494

[180] Navanandan N Hatoun J Celedón J Liu A . Predicting severe asthma exacerbations in children: blueprint for today and tomorrow. J Allergy Clin Immunol Pr. (2021) 9:2619–26. doi: 10.1016/j.jaip.2021.03.039. PMID: 33831622

[181] Chiu C Huang M . Asthma in the precision medicine era: biologics and probiotics. Int J Mol Sci. (2021) 22:4528. doi: 10.3390/ijms22094528. PMID: 33926084 PMC8123613

[182] Zou X Wu J Ye H Feng D Meng P Yang H . Associations between gut microbiota and asthma endotypes: a cross-sectional study in South China based on patients with newly diagnosed asthma. J Asthma Allergy. (2021) 14:981–92. doi: 10.2147/JAA.S320088. PMID: 34408443 PMC8367087

[183] Alswat A . The influence of the gut microbiota on host health: a focus on the gut-lung axis and therapeutic approaches. Life Basel. (2024) 14:1279. doi: 10.3390/life14101279. PMID: 39459579 PMC11509314

[184] Alcazar C Paes V Shao Y Oesser C Miltz A Lawley T . The association between early-life gut microbiota and childhood respiratory diseases: a systematic review. Lancet Microbe. (2022) 3:e867–80. doi: 10.1016/S2666-5247(22)00184-7. PMID: 35988549 PMC10499762

[185] Chunxi L Haiyue L Yanxia L Jianbing P Jin S . The gut microbiota and respiratory diseases: new evidence. J Immunol Res. (2020) 2020:2340670. doi: 10.1155/2020/2340670. PMID: 32802893 PMC7415116

